# A Refined Set of Universal Force Field Parameters for Some Metal Nodes in Metal–Organic Frameworks

**DOI:** 10.1021/acs.jctc.4c01113

**Published:** 2024-11-27

**Authors:** Yutao Li, Xin Jin, Elias Moubarak, Berend Smit

**Affiliations:** Laboratory of molecular simulation (LSMO), Institut des Sciences et Ingénierie Chimiques, École Polytechnique Fédérale de Lausanne (EPFL), Rue de l’Industrie 17, CH-1951 Sion, Switzerland

## Abstract

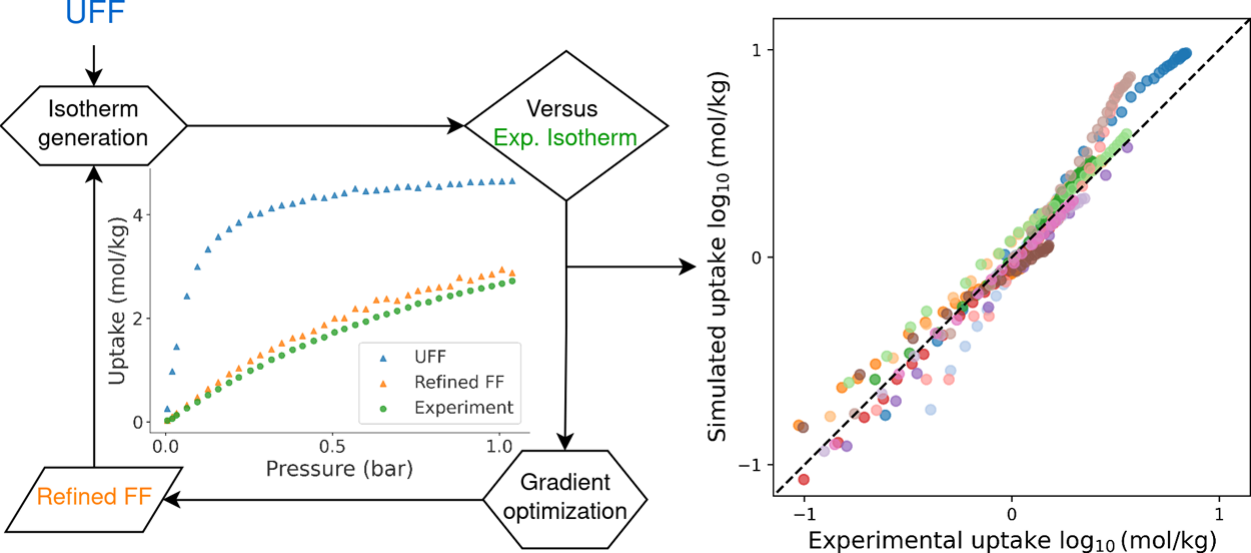

Metal–organic frameworks (MOFs) exhibit promise as porous materials for carbon capture due to their design versatility and large pore sizes. The generic force fields (e.g., UFF and Dreiding) use one universal set of Lennard-Jones parameters for each element, while MOFs have a much richer local chemical environment than those chemical environments used to fit the UFF. When MOFs contain hard-Lewis acid metals, UFF systematically overestimates CO_2_ uptakes. To address this, we developed a workflow to affordably and efficiently generate reliable force fields to predict CO_2_ adsorption isotherms of MOFs containing metals from groups IIA (Mg, Ca, Sr, and Ba) and IIIA (Al, Ga, and In), connected to various carboxylate ligands. This method uses experimental isotherms as input. The optimal parameters are obtained by minimizing the loss function of the experimental and simulated isotherms, in which we use the Multistate Bennett Acceptance Ratio (MBAR) theory to derive the functionality relationship of loss functions in terms of force field parameters.

## 1 Introduction

Metal–organic frameworks (MOFs) are a promising class of CO_2_ absorbents owing to their large capacity for the adsorption of gases and their structural and chemical tunability.^1^ Over 90,000 MOFs have been synthesized,^2^ and over a trillion structures have been generated in silico.^3^ High throughput computational screening studies have been conducted to find the optimal MOF for a given application.^4^ Such screening studies use molecular simulation to predict the adsorption properties. These simulations rely on generic force fields, such as the universal force field (UFF)^5^ or Dreiding,^6^ to describe the interactions between the adsorbed gas molecules and the atoms of the MOFs.^3,7,8^

The generic force fields use one universal set of Lennard-Jones parameters for each element. However, MOFs have a much richer local chemical environment than those chemical environments used to fit the UFF force field. Open metal sites are a well-known example where the UFF fails to capture the chemical environment accurately, typically leading to a significant underestimation of the CO_2_ loading.^9,10^ Also, for CO_2_ adsorption in fully coordinated Al-MOFs, UFF substantially overestimates the isotherms.^7^

To improve the predictions of the adsorption properties for those systems where UFF fails, researchers have refitted the force field parameters against experimental isotherms or quantum mechanical calculations for specific MOFs.^10−14^ For example, Pérez-Pellitero et al.^12^ introduced empirical scaling factors of 0.69 for ϵ_UFF_ and 0.95 for σ_UFF_ in the UFF force field to improve the predictions of the adsorption isotherms in ZIF-8 and showed that these parameters could be transferred to ZIF-76. McDaniel et al.^15^ argued that different scaling parameters could give similar results, hence questioning its transferability. McDaniel et al.^15^ developed a framework to derive a specific force field for each ZIF using ab initio calculations and showed that accurate predictions of the adsorption isotherms could be made for ZIF-8 and ZIF-71.

An alternative approach is to use accurate quantum chemical calculations to describe the interactions of a guest molecule with open metal sites, and these data are used to correct the force field.^9,10,13,14^ However, deriving an ab initio force field for each MOF is time-consuming, which makes such an approach difficult to use in screening studies.

In this work, we developed a workflow to affordably and efficiently tune force field (FF) parameters using experimental isotherms as input. We used this approach to fine-tune the UFF parameters for Al-containing MOFs. In addition, we analyzed the reasons behind the overestimation of the CO_2_ isotherms for these Al-MOFs. This analysis suggested that other metals may exhibit similar issues. We showed that our fine-tuned parameters can be transferred to MOFs that contain these metals.

## 2 Methodology

### 2.1 Workflow to Develop Force Fields

**Figure 1:**
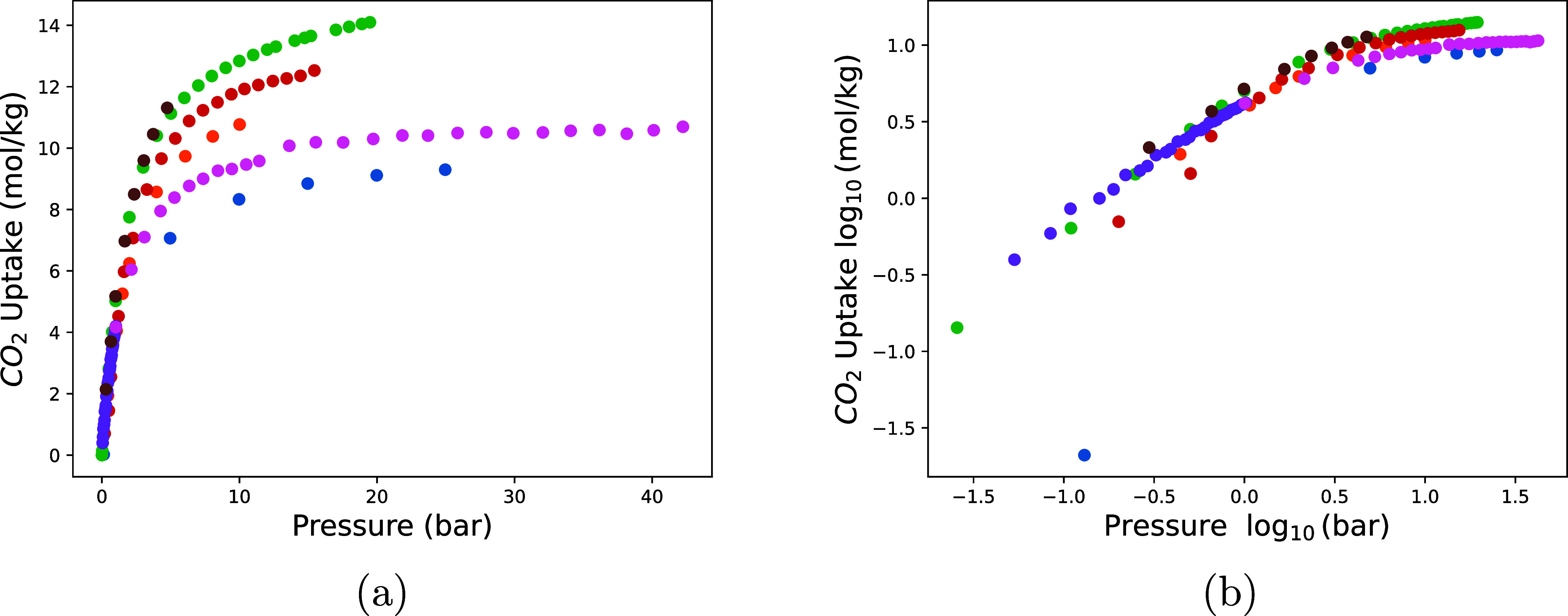
(a) CO_2_ uptake as a function of pressure for seven experimental isotherms of HKUST-1 at 298 K.^16−22^ (b) Logarithmic representation of CO_2_ uptake (log_10_ scale) as a function of pressure for the same isotherms.

The starting point of our workflow is assuming that CO_2_ uptake at low pressure is a reasonable starting point to assess the CO_2_ interactions with the MOF atoms. Crystal defects or partial activation of the MOF often impact adsorption at high pressures. To illustrate this, we collected seven experimental CO_2_ adsorption isotherms of HKUST-1 (see Figure 1). The saturation loadings show significant deviations. At the low pressures, the deviations between the experimental data sets are much smaller.

Therefore, we chose the 0 to 0.2 bar range of the CO_2_ isotherm to refine the force field. In addition, we computed the binding energy using Density Functional Theory (DFT) to validate the force field further.

**Figure 2:**
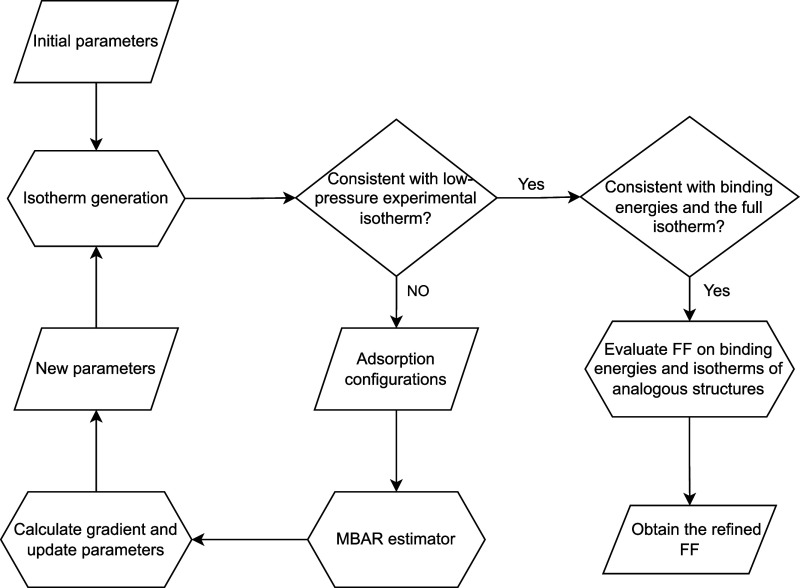
Workflow to obtain a refined force field from experimental isotherms of one MOF structure. Binding energies and experimental isotherms of analogous structures are used to validate the transferability of the refined FF.

The workflow we used to refine the force field systematically consists of the following steps (see Figure 2 for the flow diagram):

1. *Isotherm generation:* Grand Canonical Monte Carlo (GCMC)^23^ simulations are used to predict the adsorption isotherms.
2. *MBAR estimator:* Multistate Bennett Acceptance Ratio (MBAR) theory is used to derive the differentiable functionality relationship between the uptake and force field parameters.
3. *Gradient descent optimization:* The differentiation of uptakes with respect to parameters is used to update the FF.
4. *Collection of structures and isotherms:* Similar structures are found in the reported data set, and their experimental isotherms are used to validate the FF.
5. *Binding energy calculation:* binding sites of CO_2_ are searched, and binding energies are calculated from the FF and DFT. The comparison of binding energies is used to validate the FF.

In the next section, we discuss each step in detail.

### 2.2 Force Fields for Gas Adsorption within MOFs

In our GCMC simulations, we assume that the MOF framework is rigid. The interactions of the atoms in the MOFs with the adsorbed gas molecules are described by van der Waals (vdW) and Coulomb interactions shown in eq 1. The vdW interactions are described with the Lennard-Jones potential. The interactions among the gas molecules are described with the TraPPE force field.^24^ The partial charges on gas molecules are inherited from the TraPPE force field, and those on the MOF atoms are derived by the DDEC method.^25^ The Ewald summation is used to calculate the electrostatic interactions.

As the MOF is kept rigid, we only need to consider the interactions between the atoms of the MOF and the gas molecules:
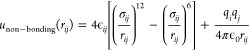
1

For the Lennard-Jones interactions, we use a cutoff distance of 12.8 Å as suggested by Jablonka et al.^26^ The entire potential is shifted based on its value at the cutoff distance to avoid the truncation error. Additionally, a tail correction is applied.^26^ The standard Lorentz–Berthelot combining rules are applied to obtain the Lennard-Jones parameters ϵ_*ij*_ and σ_*ij*_ between different types of atoms:
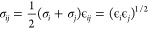
2

Previous works^11,12^ have shown that refitting the parameters resulted in significant changes in ϵ parameters, while the σ did not change much. Based on these arguments, we have deliberately restricted the parameter “search space” by keeping the σ the same as in UFF. Our rationale for fine-tuning the UFF parameters is that the chemical environments we find in MOFs can differ from those considered when the UFF parameters were optimized. One can expect that changes in the chemical environment may change the dispersive interactions but will impact the atoms’ radius much less. We use the original UFF parameter as our initial guess.

### 2.3 Structure Optimization

To ensure that our most important results can be reproduced, we implemented our workflows in AiiDA (Automated Interactive Infrastructure and Database for Computational Science^27^). AiiDA orchestrates the different steps, managing the interaction of various codes and providing automation and similarity of the calculations. The workflows are published and maintained as the *aiida-lsmo* plugin on GitHub.^28^ The methods described in the following three sections are executed through work chains in the *aiida-lsmo* plugin.

We used the MOF crystal structures from public data sets.^29,30^ These structures have already been cleaned and optimized using DFT calculations. However, two structures were missing some hydrogen atoms. For these structures, we added missing hydrogen atoms manually and used DFT calculations to optimize the MOF structure. Both geometry optimization and cell optimization were performed sequentially, implemented as the *Cp2kMultistageDdec* workchain in the *aiida-lsmo* plugin.^31^

The partial charges on the MOF atoms are derived by the DDEC method from a DFT calculation,^25^ which is also included in the *Cp2kMultistageDdec* work chain. DFT calculations were performed using the Perdew–Burke–Ernzerhof (PBE) functional within the generalized gradient approximation (GGA),^32^ with van der Waals (vdW) interactions included through Grimme’s D3 corrections.^33^ The quickstep code of the CP2K package was used.^34^ We have listed the modified structures and provided the corrected CIF files in Section S2 of the SI.

### 2.4 Isotherm Generation

The adsorption isotherms were simulated in the Grand Canonical ensemble. Here, 15,000 cycles were used for equilibration and 15,000 cycles for production. Simulations at subsequent pressure points were performed starting from the restart file of the previous pressure step, thus reducing the number of cycles necessary for initialization. The process was done by the *Isotherm* work chain in the *aiida-lsmo* plugin.^31^ The RASPA molecular simulation software^35^ was used to perform the GCMC simulations.

### 2.5 Binding Energy Calculation

The *BindingSite* work chain in the *aiida-lsmo* workflow^31^ was used to identify the minimum energy configuration for a single gas molecule (e.g., CO_2_, N_2_, and CH_4_) in the MOFs, from which we obtained the binding energies.

This workflow utilizes a series of *NV T* Monte Carlo simulations, each consisting of 10,000 steps, gradually lowering the temperature from 300 K down to 50 K in steps of 50 K. The configuration with the lowest energy from this simulated annealing step is then used in an energy minimization step where we fine-tune the CO_2_ configuration until we have found the minimal energy configuration. We assume the MOF is rigid in these calculations. To compute this minimum energy configuration, we take the CO_2_ configuration with the lowest energy and perform energy optimization to find the optimal position of the adsorbed molecule. This is done both with the force field and with DFT. Here, we use the same DFT calculation settings for the structure optimization.

### 2.6 Differentiable Optimization

We use an iterative procedure to find the optimal force field parameters, comparing the simulated isotherm with experimental ones. If these two deviate more than our set tolerance, we compute as a loss function:
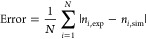
3In this loss function, *n*_exp_ and *n*_sim_ are gas uptakes from the experimental and simulated isotherms, respectively. The *N* points for which we compute the loss function are in the pressure range from 0 to 0.2 bar.

Typical minimization routines estimate the next step in the iteration from the derivative of the loss function with respect to the parameters that need to be optimized. This procedure is repeated until the loss function is smaller than the desired tolerance. A technical difficulty is that differentiation of uptakes with respect to force field parameters is a nontrivial task because there is no well-differentiable functionality relationship between uptake and force field parameters. Multistate Bennett Acceptance Ratio (MBAR) theory can be used to derive the functionality relationship between uptake and force field parameters.

The MBAR theory estimates the ensemble average of properties *A* of a system in which the adsorbed molecules interact with a potential *U*_2_ from trajectories of a system in which the particles interact with a (slightly) different potential *U*_1_.^36^ The ensemble average of *A* is defined as
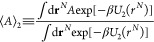
4This equation can be rewritten as an ensemble of particles interacting with a potential *U*_1_:
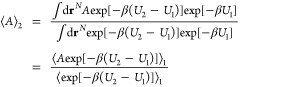
5where the subscript in the ensemble average indicates which system is simulated. In practice, the MBAR theory only gives sufficiently accurate results if there is sufficient overlap between the two different ensembles, i.e., the configuration sampled by system 1 also contributes significantly to system 2.

This assumption is nontrivial but works in our example. For force field optimization, the only difference between *U*_1_ and *U*_2_ is that the old parameters are used in *U*_1_, and *U*_2_ gives the estimate of our *A* for our model with a new set of force field parameters. The change in force field parameters is not large because the gradient descent optimization is used to update parameters. Therefore, we can safely assume we have sufficient overlap for MBAR theory to work well. We can now compute derivatives of the loading in terms of ϵ, *f* (ϵ). We used automatic differentiation to calculate the derivatives.^37^

We can represent *f*(ϵ) as a composition of a series of functions:
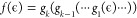
6where *g*_*i*_ is a function that maps an intermediate variable to the next intermediate variable *y*_*i*+1_. According to the chain rule, the gradient with respect to ϵ can be expressed as
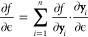
7Each term can be further broken down:
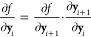
8This process can decompose a complex function into a series of simpler functions, forming a computational graph. In this graph, gradients can be calculated by symbolic differentiation and are propagated backward to compute the gradient of the loading with respect to ϵ.

Using eq 5, we can build the functionality relationship in terms of force field parameters and calculate the derivative. Because loss functions are just the difference between experimental and simulated isotherms in the low-pressure regions, it is easy to calculate the derivative of loss functions in terms of force field parameters. The calculated gradients are used to update the force field parameters. For the implementation in our code, we use the package DMFF.^37^

### 2.7 Collection of Structures and Experimental Isotherms

To refit the parameters, we used the experimental data sets provided by the PrISMa project^29^ and a data set derived from the CoRE MOF database,^38^ which has been curated by Jablonka et al.^30^ In our fitting, we focused on the low-pressure region. Specifically, we selected the first three points from the experimental isotherm of CAU-10 where the pressures are below 0.2 bar.

## 3 Results and Discussion

### 3.1 Force Field Development for Al-MOF

**Figure 3:**
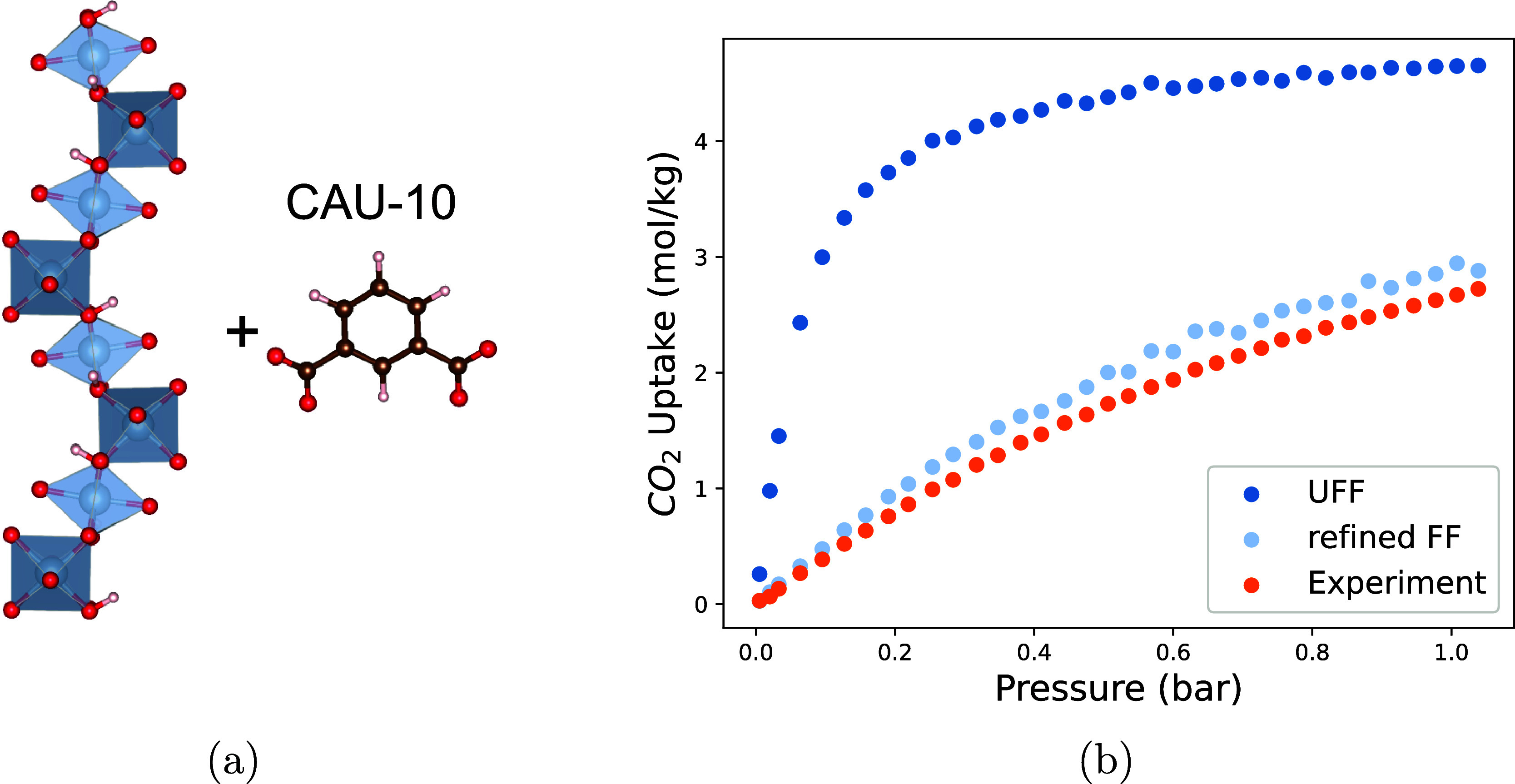
(a) CAU-10 is made up of 4-fold helical chains of cis-corner-sharing AlO_6_ octahedra and ligands between chains (red: oxygen, blue: aluminum, brown: carbon, and white: hydrogen), (b) comparison between experimental isotherms (296 K) of CAU-10(Al)^40^ and simulated isotherms as generated by UFF and the refined force field.

CAU-10 is a typical example of a fully coordinated Al-MOF. CAU-10 has Al^3+^ as metal node and benzene-1,3-dicarboxylate as linker. The framework structure of CAU-10 shown in Figure 3a is composed of 4-fold helical chains of cis-corner-sharing AlO_6_ octahedra, which are interconnected by the ligands.^39^

**Figure 4:**
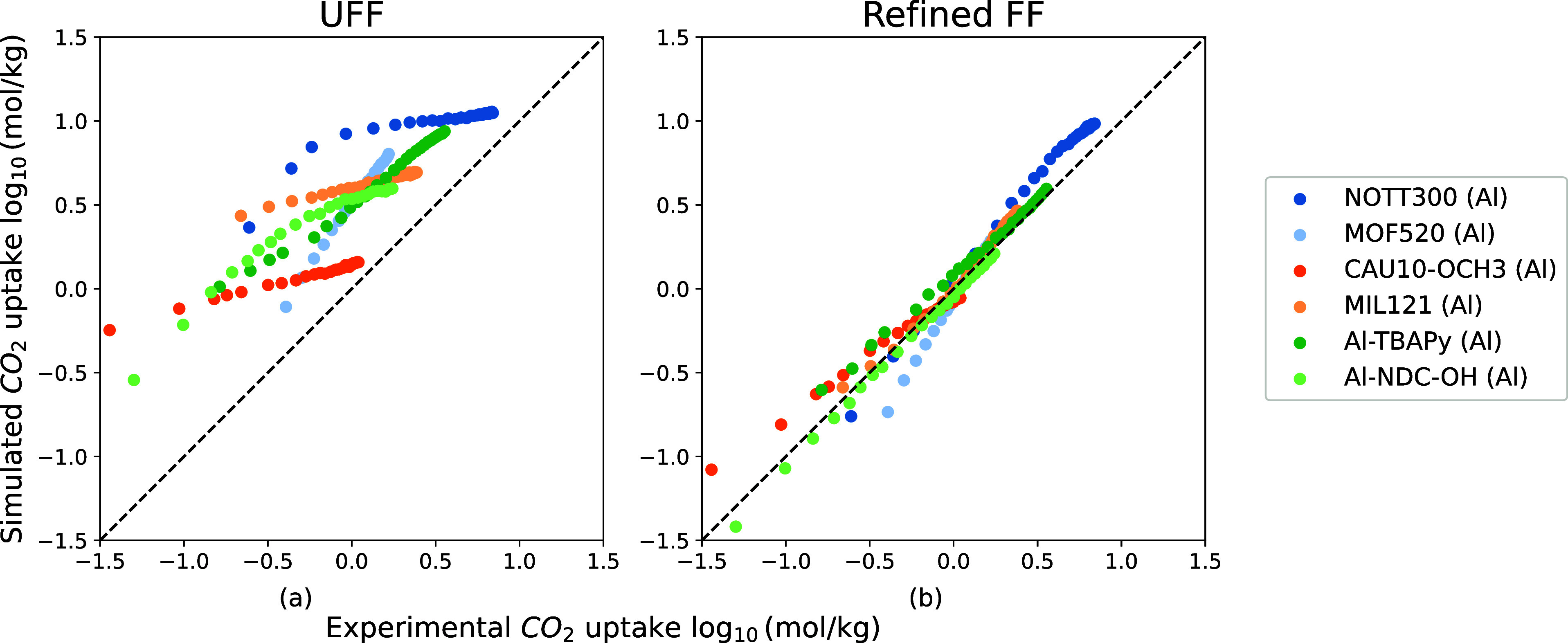
Comparison between six experimental CO_2_ isotherms of Al-MOFs (NOTT-300,^43^ MOF-520,^44^ CAU-10-OCH_3_,^45^ Al-TBAPy,^7^ Al(NDC)(OH),^46^ MIL-121^47^) and simulated ones from UFF and the refined FF. The pressures of experimental isotherms range from 0 to 2 bar and their temperatures from 273 to 313 K. A logarithmic scale is used to compare isotherms.

In Figure 3b, we compare the experimental CO_2_ isotherms with the one predicted from UFF in CAU-10. This comparison is a typical example of the issue we aim to address: UFF significantly overestimates experimental loading for MOFs containing Al. Figure 4a shows that UFF also systematically overestimates the CO_2_ uptake in other MOFs containing Al.

The logical first step in our refitting strategy is to adjust the parameters of Al. However, when we set the interactions of CO_2_ with Al as zero, UFF still overestimates the experimental isotherms of Al-MOFs (see Figure S2 in the SI). We further expanded the fitting range to the entire metal cluster, i.e., optimizing both ϵ_Al_ and ϵ_O_. We could reproduce the experimental isotherms only with values for ϵ_Al_ and ϵ_O_ close to zero (see the parameters in Section S1.2 of the SI). Compared with the original values in UFF, the ϵ_O_ decreased by 93.7%, and the ϵ_Al_ decreased by 98.0%. These very low values of the interaction parameters with the metal cluster suggest that UFF not only overestimates the interactions of CO_2_ with the metal cluster but also with its ligands.

The Lennard-Jones parameters of C in UFF are inherited from a force field within polyethylene,^41^ whose carbons are sp^3^-hybridized. However, all carbons in CAU-10 are sp^2^-hybridized, which belong to aromatic rings or carboxyl groups. Since sp^3^-hybridized carbons have higher polarizability than sp^2^-hybridized carbons, one can expect a higher ϵ value for sp^3^-hybridized carbons. UFF does not have separate parameters for sp^2^-hybridized and sp^3^-hybridized carbons. Indeed,^42^ derived for C_60_ ϵ_sp2–C_/*k*_B_ = 34.9 K, which is indeed lower than the UFF value (ϵ_sp3–C_/*k*_B_ = 52.8 K). Both force fields have almost the same values for σ_C_, i.e., σ_sp2–C_ = 3.43 Å and σ_sp3–C_ = 3.39 Å, respectively.

When calculating the derivative of the loss function (eq 3) with respect to ϵ, we observed that the derivatives for C and O were more than five times higher than those for Al. This suggests that we only need to optimize ϵ_C_ and ϵ_O_, and we can keep the UFF parameters for Al.

Following the workflow outlined in Figure 2, we obtained as new force field parameters: ϵ_O_/*k*_B_ = 4.1 K and ϵ_C_/*k*_B_ = 34.7 K. As illustrated in Figure 3b, the new force field accurately reproduces the entire isotherm of CAU-10(Al). In addition, the binding energy from the refined FF is −31.6 kJ mol^–1^, which is close to −30.77 kJ mol^–1^ from DFT calculations. The binding energy from UFF is −38.2 kJ mol^–1^, which overestimates the interaction between CO_2_ and CAU-10.

### 3.2 Transferability: Al-MOFs

To test if the new force field parameters can be transferred to all Al-MOFs, we compared the predicted isotherms with the experimental isotherms for six MOFs we used as a validation set. These six MOFs contain only C, H, O, and Al, and feature aromatic rings that are benzene or fused benzene rings. Figure 4 shows that our new force field shows a significant improvement in the predictions of the experimental CO_2_ isotherms compared with UFF.

**Figure 5:**
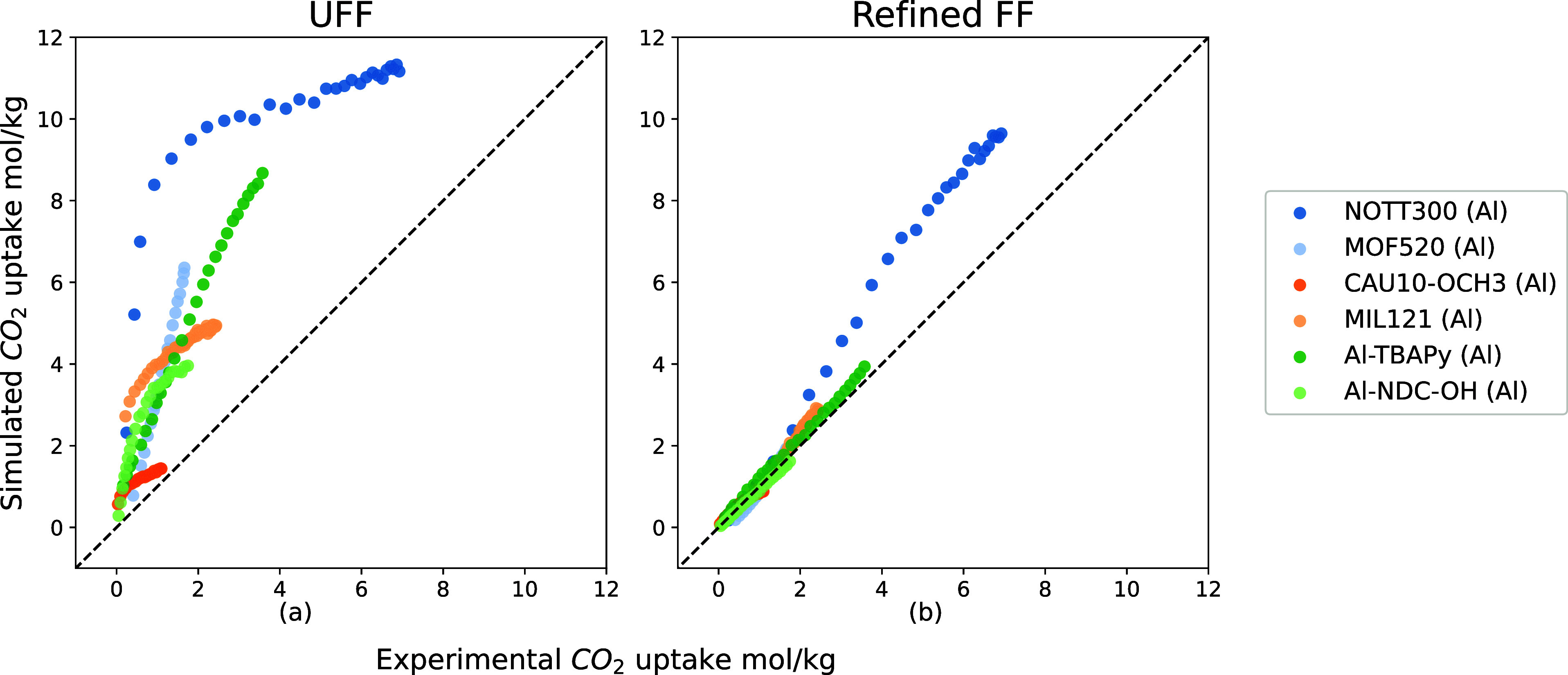
Comparison between six experimental CO_2_ isotherms of Al-MOFs (mentioned in Figure 4) and simulated ones from UFF and the refined FF. Different from Figure 4, a linear scale is used to compare isotherms.

Our refined force field provides a good description of the low-pressure region. However, we do observe differences at high loadings (see Figure 5b). In our simulations, we assume a perfect crystal, which is experimentally nearly impossible to synthesize. Hence, we would expect to overestimate the saturation loading. However, in CAU-10-OCH_3_, the simulated loading is lower than the experimental loading. Its channels are relatively narrow, and the rotation of the OCH_3_ groups in the ligands could increase the accessible volume for CO_2_ adsorption. In our GCMC simulations, however, we assumed rigid frameworks, which may cause the observed underestimation of the saturation loading of CO_2_ in CAU-10-OCH_3_ (see Figure S5 in the SI).

**Figure 6:**
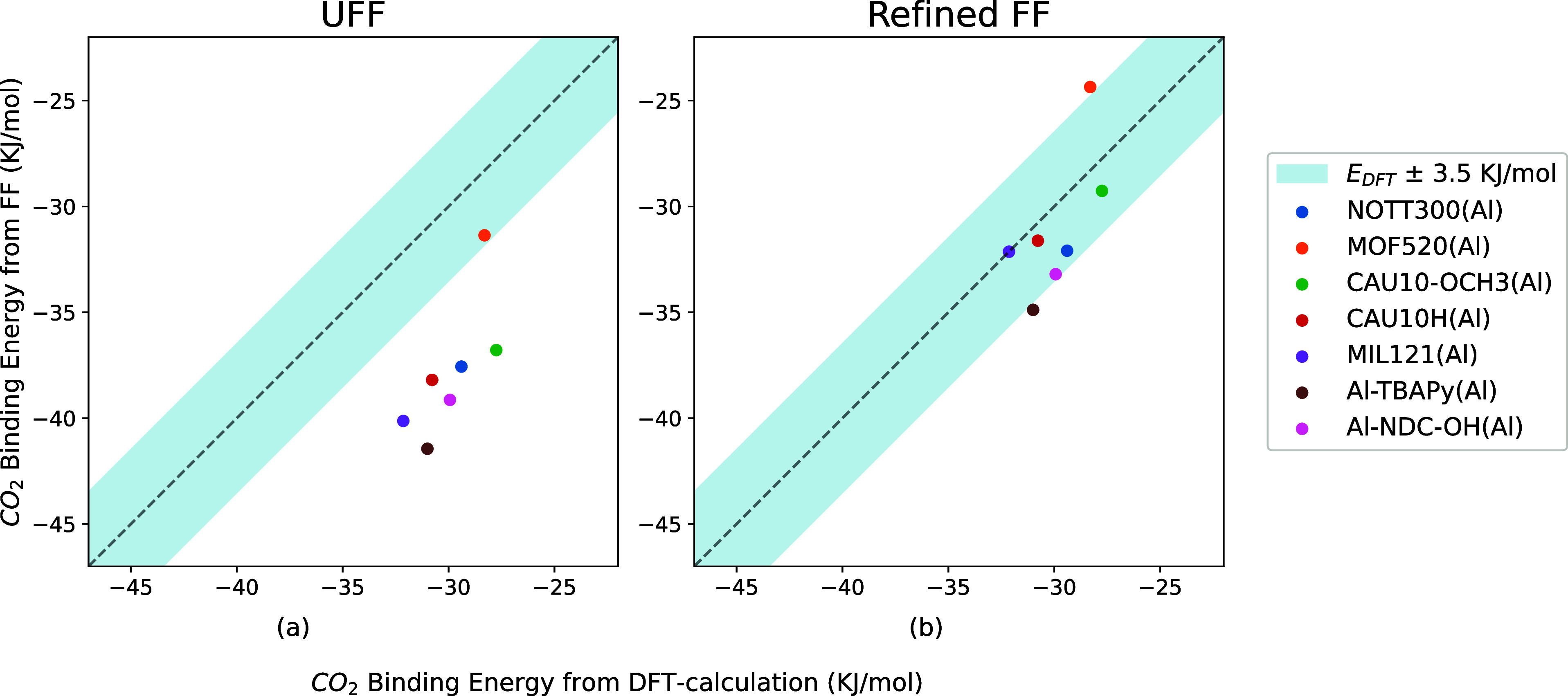
Comparison among binding energies of seven Al-MOF structures including CAU-10, which were calculated from DFT, UFF, and the refined FF.

It is interesting to compare the binding energies from UFF and our refined force field with the binding energy from DFT. As expected, Figure 6a shows that UFF systematically overestimates binding energies. The binding energies from our refined force field are consistent with the DFT calculations, whose deviations are below 3.5 kJ mol^–1^ for MOFs containing C, H, O, and Al. Due to the different shapes of the potential energy surfaces (PES) in DFT calculations and force field (FF) methods, their binding energies should have a theoretical deviation. Here, we consider 3.5 kJ mol^–1^ as a tolerance standard, indicating that an FF provides reliable binding energies.

One can also question how unique our set of parameters is. In Section S1.3 of the SI, we compare different sets of ϵ_O_ and ϵ_C_, which we obtain from different ways of optimizing these parameters. This comparison shows that the selected set of parameters is a good compromise between accuracy and transferability.

### 3.3 Ion Polarization Effect

In UFF, Lennard-Jones parameters are derived from elements or simple complexes that do not involve metal–oxygen coordination. Therefore, the ion polarization between metal ions and oxygen is not considered. When oxygen is strongly polarized by metal ions, its electronic distribution is deformed, and the polarizability decreases significantly. However, the electronic distribution in metal ions remains relatively unchanged, and their polarizabilities are mainly unaffected.

UFF does not consider the ion polarization between metal ions and oxygen, which is particularly serious for MOFs containing hard-Lewis acid metals. Due to their low radius and high charges, they have strong polarization power, and the polarizability of oxygen coordinated with them decreases. If this hypothesis is correct, a consequence is that UFF should also systematically overestimate gas adsorption for MOFs containing other metals in groups IIA and IIIA. To test this hypothesis, we collected experimental adsorption data from MOFs containing these metals (In, Ga, Mg, Ca, Sr, and Ba).

### 3.4 Transferability: M-MOF(M = In, Ga)

Due to the varying polarization effects of different metals on oxygen, we may need to refit the ϵ_O_. We use the same values as in Al-MOFs for carbon as long as the ligands are similar. We use the same strategy to refit ϵ_O_ within MIL-68(In). And we obtain an ϵ_O_/*k*_B_ of 5.1 K.

It is so close to 4.1 K in Al-MOFs that the two refined FFs can generate almost the same simulated isotherms for MIL-68(In) (see Figure S6 in the SI). Therefore, we assume that metal ions in group IIIA (i.e., Al^3+^, In^3+^, Ga^3+^) can share the same ϵ_O_ because they have the same +3 charges. If the hypothesis is correct, the refined FF derived from Al-MOFs can be transferred to MOFs containing In and Ga.

**Figure 7:**
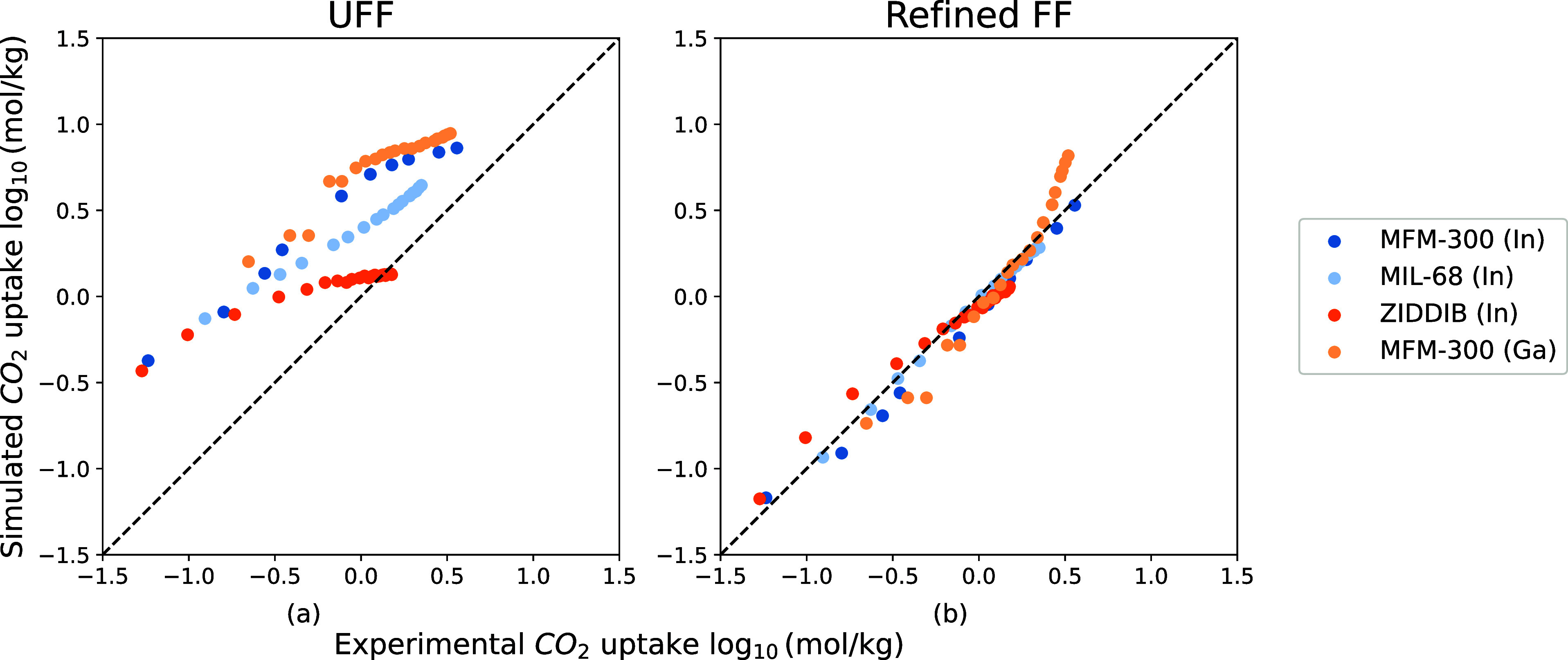
Comparison between 4 experimental CO_2_ isotherms of M-MOFs (M = In, Ga) and simulated ones from UFF and the refined FF. The pressures of experimental isotherms range from 0 to 1 bar and their temperatures from 273 to 313 K. A logarithmic scale is used to compare both low-pressure and high-pressure regions. The figure shows experimental CO_2_ isotherms of MFM-300(In),^48^ MIL-68(In),^49^ In-PDC (CCDC identifier: ZIDDIB),^50^ and MFM-300(Ga).^51^

Figure 7 compares experimental CO_2_ uptakes with simulated uptakes predicted by UFF and our refined FF. Figure 7a shows that UFF systematically overestimates the CO_2_ loadings for these MOFs. Our new force field shows a significant improvement in the predictions of the experimental CO_2_ isotherms (see Figure 7b).

**Figure 8:**
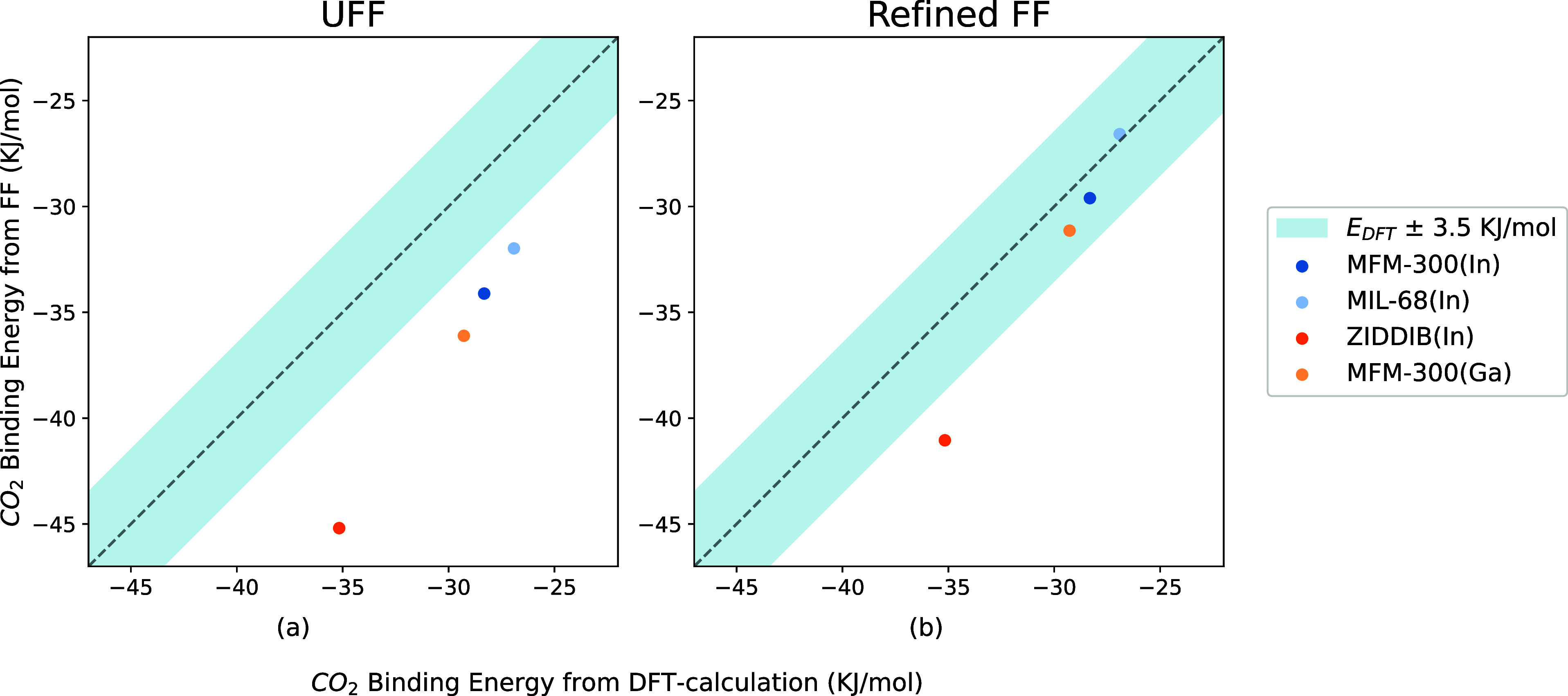
Comparison among binding energies of 4 M-MOFs (M = In, Ga), which was calculated from DFT, UFF, and the refined FF.

Figure 8a shows that UFF systematically overestimates the binding energies. The binding energies from our refined force field are consistent with the DFT calculations except ZIDDIB(In). ZIDDIB(In) is the only structure in the series with an aromatic heterocycle (pyridine). Our refined FF can reproduce the experimental isotherms but gives a relatively high CO_2_ binding energy of −41.04 kJ mol^–1^ compared to −35.16 kJ mol^–1^ from the DFT calculation. However, the refined FF is still closer to DFT than UFF (−45.19 kJ mol^–1^).

### 3.5 Force Field Development For Mg-MOF

The adsorption of CO_2_ for MOFs with metals from group IIA (i.e., Mg, Ca, Ba, and Sr) is also overestimated by the UFF force field. We use the same strategy to refine the UFF for metals in group IIA.

**Figure 9:**
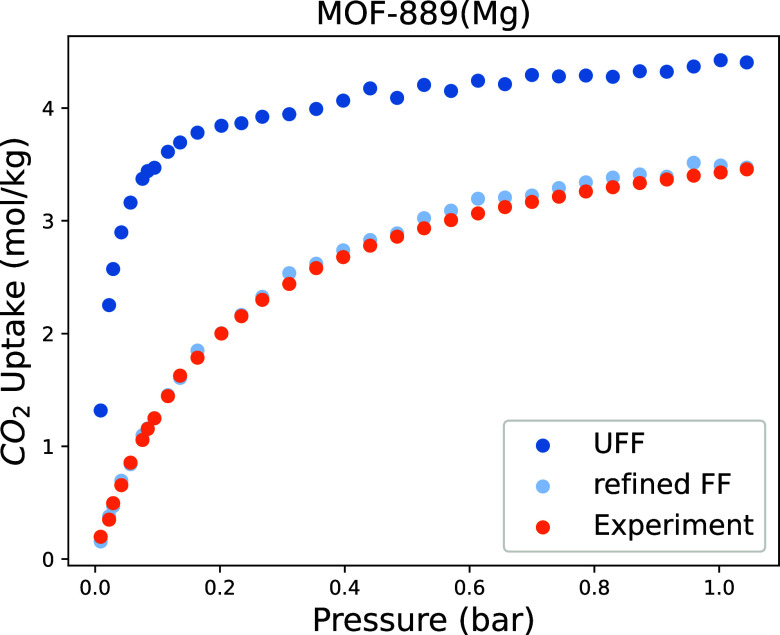
Comparison between MOF-889(Mg) experimental isotherm^52^ at 273 K and simulated isotherms generated by UFF and refined force fields.

MOF-889 consists of Mg^2+^ connected by H_6_CPB(1,2,3,4,5,6-hexakis(4-carboxyphenyl) benzene). In Figure 9, we compare its CO_2_ adsorption isotherm as predicted by UFF with the experimental one. Also, in this case, UFF significantly overestimates the CO_2_ uptake. We used a similar procedure to refit ϵ_O_ for metals in group IIA. The parameter (ϵ_O_/*k*_B_ = 11.9 K) was refined using four points from the experimental isotherm at pressures ranging from 0 to 0.1 bar. Figure 9 shows that our refined parameters reproduce the complete experimental isotherm.

The CO_2_ binding energy of MOF-889, determined through DFT calculations, is −35.69 kJ mol^–1^, which aligns closely with the −35.05 kJ mol^–1^ from our refined force field. In contrast, the binding energy from UFF is much larger, i.e., −41.02 kJ mol^–1^.

### 3.6 Transferability: M-MOF(M = Mg, Ca, Sr, and Ba)

**Figure 10:**
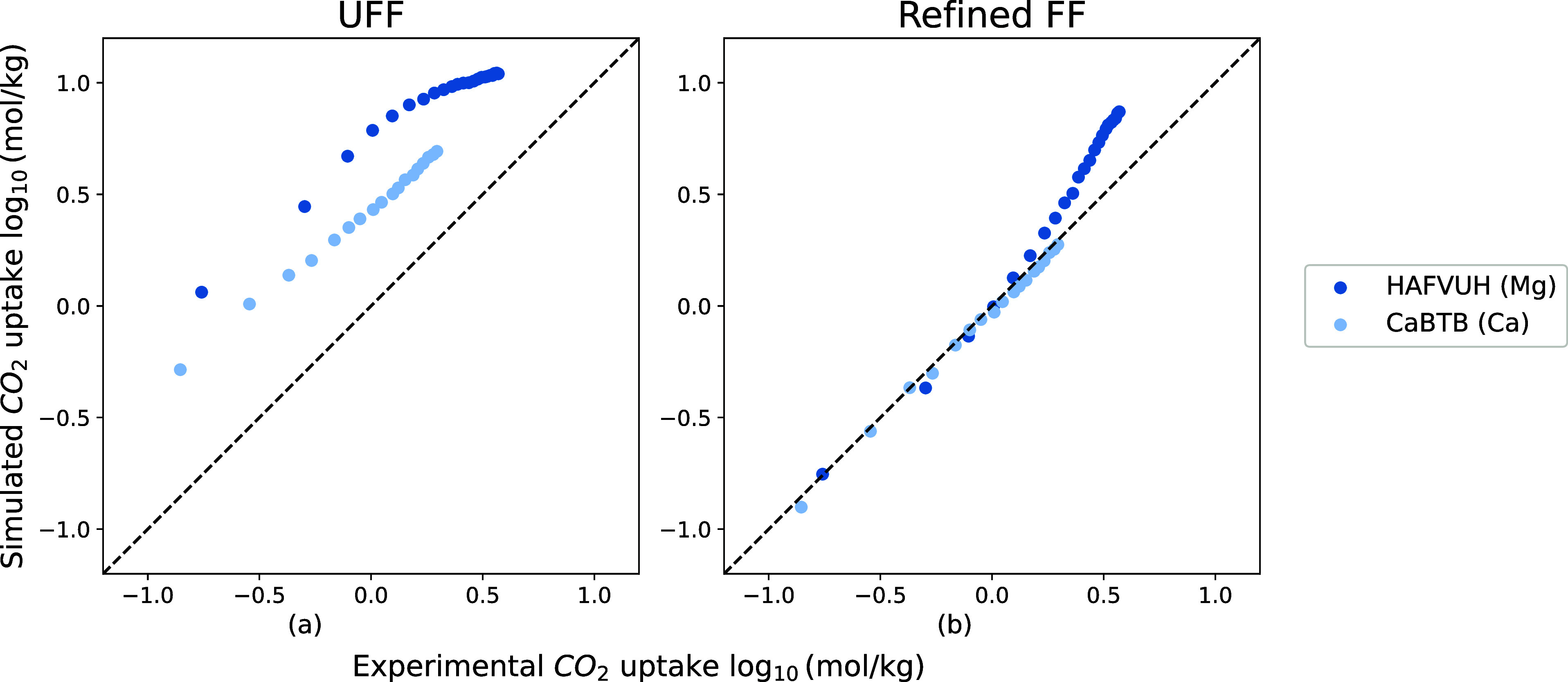
Comparison between 2 experimental isotherms of M-MOFs (M = Mg, Ca) and simulated ones from UFF and the refined FF. The two MOFs are HAFVUH^53^ and Ca-BTB.^54^

To test the transferability of our refined force field, we compared our predicted isotherms with experimental ones in metals of group IIA. In Figure 10, we compare the refined FF with the experimental CO_2_ isotherms of Ca-BTB and HAFVUH. Figure 10b shows that also, for these MOFs, our new force field provides an accurate description.

**Figure 11:**
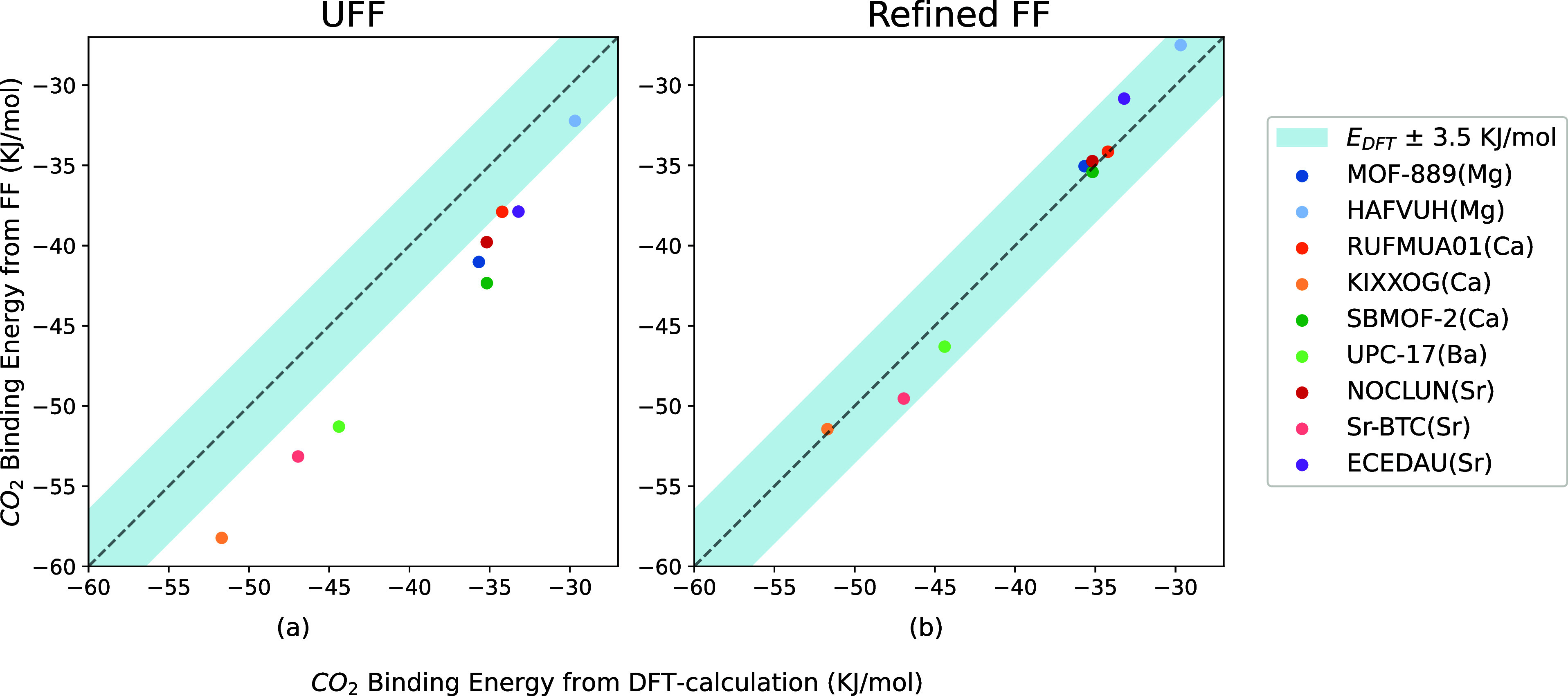
Comparison among binding energies of 9 M-MOF (M = Mg, Ca, Sr, and Ba) structures calculated from DFT, UFF, and the refined FF. The MOF structures are Ca-BTC (CCDC identifier:RUFMUA01),^55^ Ca-1,4-NDA(CCDC identifier:KIXXOG),^56^ SBMOF-2 (Ca),^57^ Sr-4,4-dicarboxylic acid diphenylacetylene (CCDC identifier: ECEDAU),^58^ Sr-BTC,^59^ Sr-TPA,^60^ and UPC-17(Ba).^61^

There are many more Mg-, Ca-, Ba-, or Sr-MOFs for which we expect similar deviations. However, we could not find additional experimental CO_2_ isotherms. We can compare the FF predictions of the binding energies for these MOFs with those obtained from DFT. Figure 11a shows that UFF overestimates the binding energies compared with DFT results. In contrast, the binding energies calculated from our refined FF are consistent with DFT results. This comparison of the binding energies for these nine structures further validates that the refined FF can be transferred to other metals in group IIA.

Metal ions in group IIIA have +3 charges, which are usually harder Lewis acids than those in group IIA. This suggests that UFF should also overestimate the binding energies of CO_2_ in MOFs containing metals of group IIA. This is indeed what we observed. In Figures 6, 8, and 11, the average deviations of binding energies from UFF and DFT are 7.64 and 5.3 kJ mol^–1^ for metals in groups IIIA and IIA, respectively. In our refined FF, oxygen connected to metals in group IIIA has a lower ϵ_O_ than those connected to metals in group IIA.

### 3.7 Transferability: Nitrogen and Methane

We developed a transferable FF for MOFs containing metals in groups IIA and IIIA from their CO_2_ experimental isotherms. It is important to see if our parameters can also be used for other gases, such as N_2_ and CH_4_. In addition, in carbon capture or natural gas sweetening, one has to separate CO_2_ from N_2_ and CH_4_, respectively.

**Figure 12:**
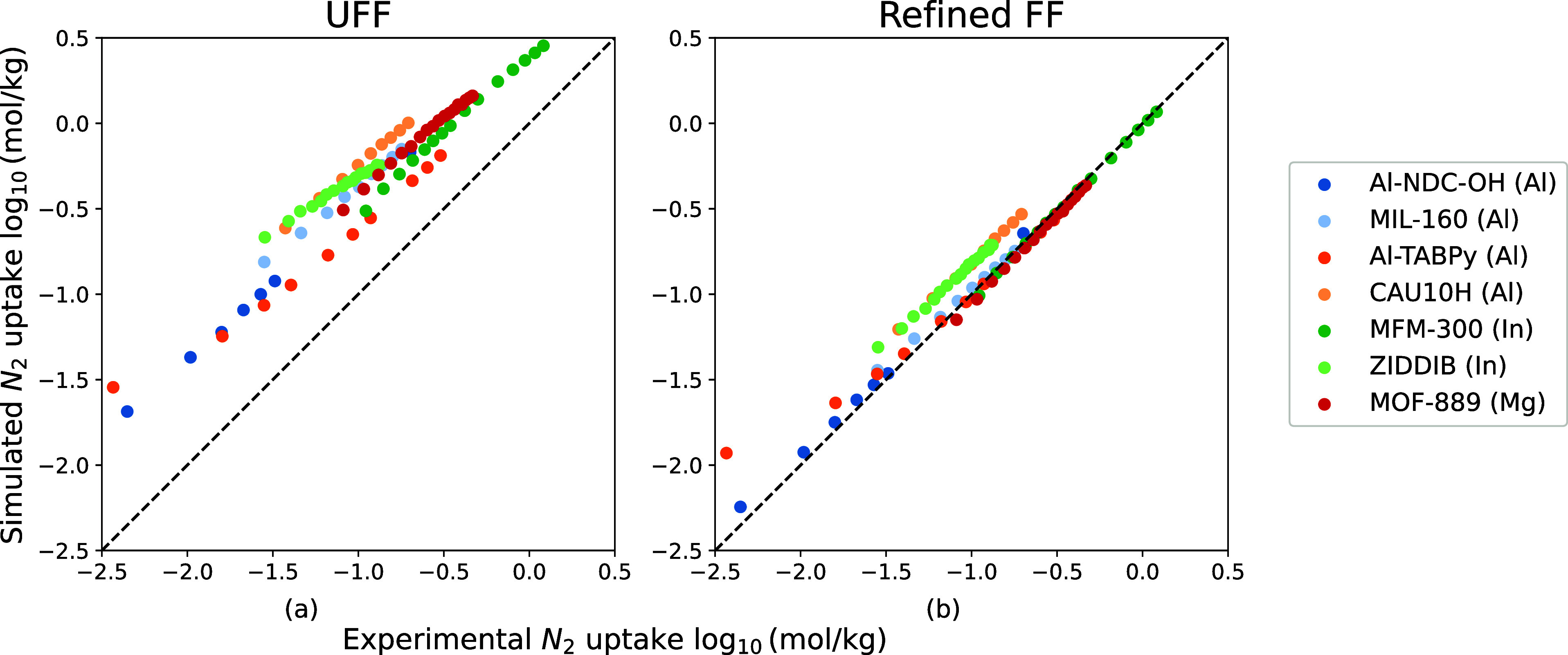
Comparison between seven experimental N_2_ isotherms of M-MOF (M = Mg, Al, In) and simulated ones from UFF and the refined FF. The experimental structures are MIL-160(Al),^62^ CAU-10(Al),^62^ Al-TBAPy(Al),^63^ Al-NDC–OH(Al),^46^ MFM-300(In),^48^ ZIDDIB(In),^50^ and MOF-889 (Mg).^52^

**Figure 13:**
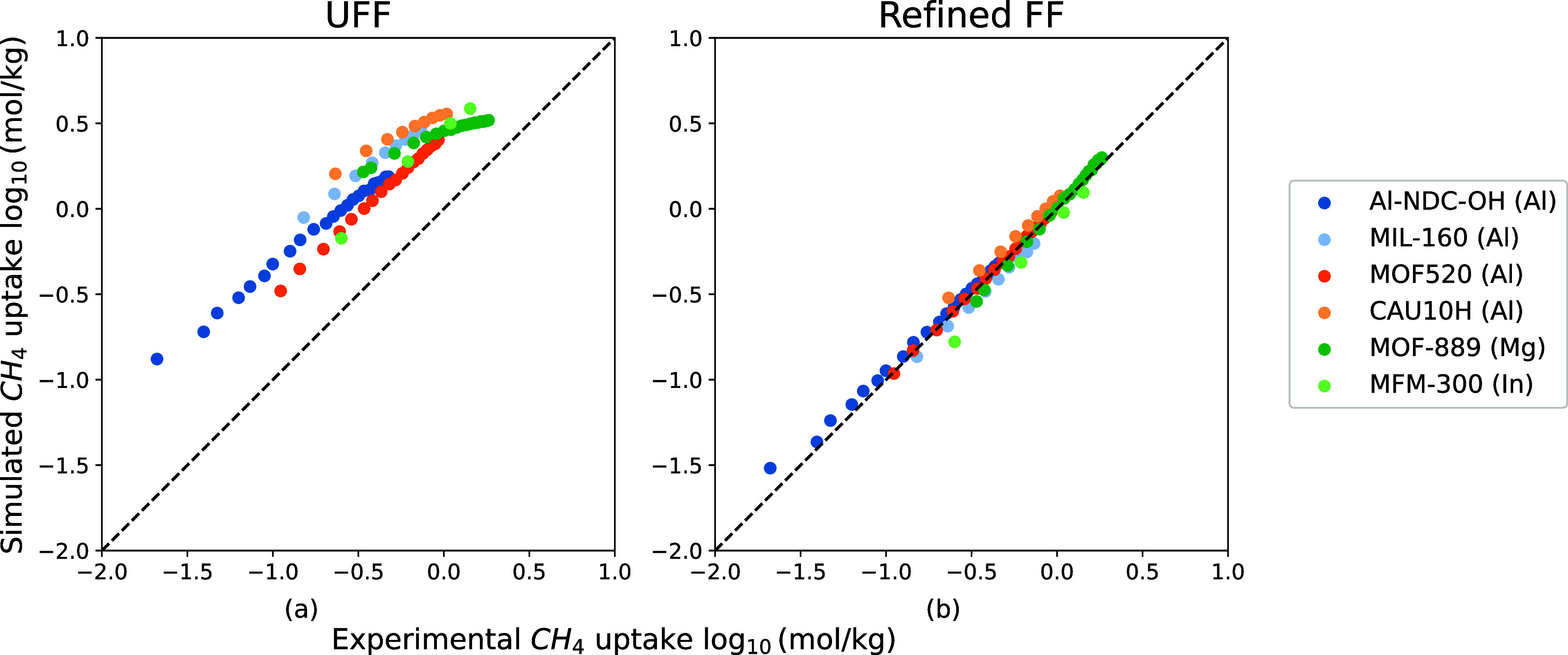
Comparison between six experimental CH_4_ isotherms of M-MOF (M = Mg, Al, In) and simulated ones from UFF and the refined FF. The experimental structures areMIL-160(Al),^62^ CAU-10(Al),^62^ MOF-520(Al),^64^ Al-NDC–OH(Al),^46^ MFM-300(In),^48^ and MOF-889 (Mg).^52^

We compare the predictions of our force field with seven experimental N_2_ isotherms and six experimental CH_4_ isotherms. Figures 12a and 13a show that UFF overestimates N_2_ and CH_4_ uptakes for MOFs containing metals in groups IIA and IIIA, respectively. In Figures 12b and 13b, our new force field reproduces experimental N_2_ and CH_4_ isotherms. It is expected that the force field derived from CO_2_ experimental isotherms can be transferred to other gas adsorption given that we only update the parameters of the MOF frameworks. In this case, we can use N_2_ and CH_4_ experimental adsorption isotherms, on top of binding energies, to validate the refined force field for CO_2_, which reduces the difficulty of obtaining experimental isotherms.

### 3.8 Force Field Development for MOFs with Furan or Pyridine

By refitting the ϵ of oxygen connected to metals from groups IIA and IIIA, we can improve the accuracy of UFF on the interactions between gas molecules and metal clusters. In addition, we had to refine ϵ_C_/*k*_B_ from 52.8 to 34.7 K because ϵ_C_ in UFF was developed for sp^3^-C and most C atoms in our examples are sp^2^-hybridized. Our refined ϵ_sp2–C_ is similar to the reported value for sp^2^-C in C_60_^42^ (i.e., ϵ_sp2–C_/*k*_B_ = 34.9 K).

Our new FF provides a new parameter for sp^2^-hybridized carbon and two new parameters for oxygen within MOFs containing metals in group IIA and group IIIA respectively. When MOFs contain metals in group IIIA, we set ϵ_C_/*k*_B_ and ϵ_O_/*k*_B_ to 34.7 and 4.1 K, respectively. And when metals belong to group IIA, we keep ϵ_C_/*k*_B_ fixed and use ϵ_O_/*k*_B_ = 11.9 K due to the different strengths of the ion polarization effect. When MOFs contain multiple metals, ϵ_O_ depends on which kinds of metal they are coordinated to.

In CAU-10-OCH_3_, some oxygen atoms belong to the linker (OCH_3_) and are not connected to Al. In this case, if we use UFF or the refined FF for oxygen of OCH_3_, we get similar CO_2_ isotherms (see Section S3.1 in the SI). This implies that we can use one parameter for one element in our examples.

However, our parameters may not work for MOFs with a chemical environment very different from what we have considered in this work. We have shown that a good indication of such a problem is that the DFT binding energy differs significantly from the one obtained with our FF (ZIDDIB(In) in Figure 8b). For those cases, we provide an affordable workflow to obtain new parameters. To illustrate this point, we choose MIL-160 as an example. It is isostructural with CAU-10, differing only in their ligands: in MIL-160, the benzene ring in CAU-10 is replaced by a furan ring.

**Figure 14:**
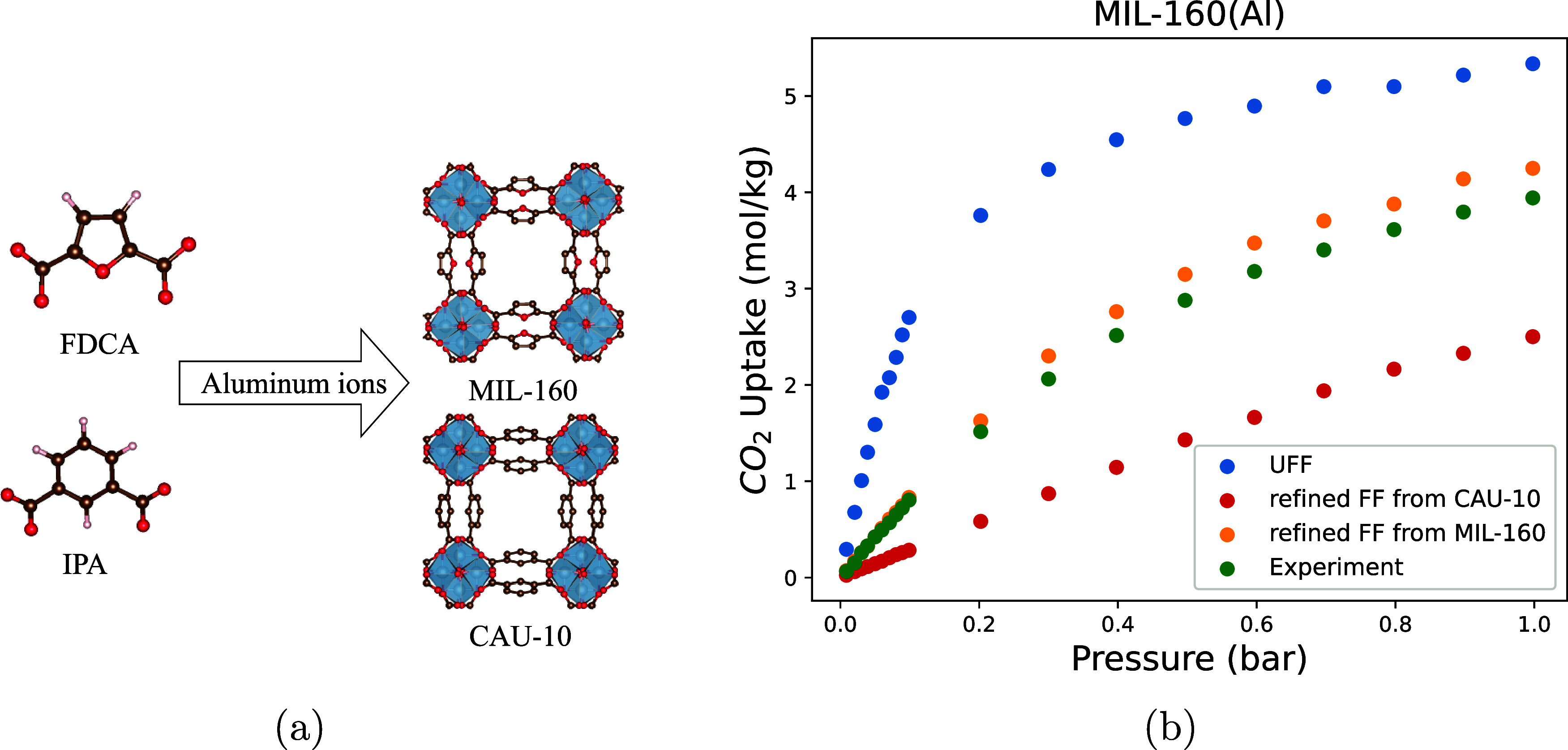
(a) MIL-160 is isostructural with CAU-10, with the same topology and metal cluster. The only difference is the ligands in CAU-10 are isophthalic acid (IPA), and MIL-160 uses 2,5-Furandicarboxylic acid (FDCA). Hydrogen (white), carbon (brown), oxygen (red), and aluminum (blue). Hydrogen atoms are ignored in MIL-160 and CAU-10. (b) Comparison of CO_2_ isotherms in 298 K from experiment and simulation using different force fields.

We compare the predictions of the CO_2_ adsorption isotherms from UFF and our refined FF from CAU-10 (see Figure 14). Figure 14 shows both UFF and our refined FF from CAU-10 can not reproduce the experimental isotherm of MIL-160. The CO_2_ binding energy in MIL-160 calculated from DFT is −32.78 kJ mol^–1^, which is significantly lower than −37.32 kJ mol^–1^ from UFF. The binding energy from the refined FF from CAU-10 is −30.69 kJ mol^–1^, which slightly underestimates the interaction between CO_2_ and the MIL-160 framework. It is consistent with the simulated isotherms shown in Figure 14. The refined FF from CAU-10 underestimates CO_2_ uptake within MIL-160.

To address these differences, we further refit ϵ_C_ for carbon in furan rings. We do not refit ϵ_O_ because most oxygen atoms still belong to the carboxyl group or coordinate to Al. We selected the first three points from the experimental isotherm of MIL-160, whose pressures range from 0 to 0.1 bar. We refit ϵ_C_ for carbon in 2,5-furandicarboxylic acid (FDCA). The new refined FF (ϵ_C_/*k*_B_ = 53.8 K) now reproduces the entire isotherm (see Figure 14). The binding energy calculated from the new refined FF is −33.64 kJ mol^–1^, which agrees well with the DFT value −32.78 kJ mol^–1^. However, since this refined ϵ_C_ for MIL-160 has not been validated for other MOFs containing furan rings, it should be used cautiously.

If we assigned a unique parameter to the carbon atoms in the furan ring, we successfully extended the refined force field to furan-containing MOFs. For MOFs containing pyridine like ZIDDIB(In), ϵ_C_/*k*_B_ = 34.7 K still can reproduce its experimental isotherms shown in Figure 7. So, we do not need to reassign ϵ_C_ in pyridine. When researchers meet MOFs containing other local environments for carbon, a similar workflow can be done to extend the refined FF to a new class of MOFs.

When encountering significant changes in the local chemical environment, such as the presence of heterocyclic structures, we can use the same workflow to refit parameters. This refinement primarily addresses shifts in polarizability within new chemical environments compared to those used in the Universal Force Field (UFF). Generally, a higher polarizability corresponds to a higher ϵ value, a trend observed in our examples.

For carbon atoms, the sp^3^-hybridized carbons exhibit less s-character, resulting in an electron density that is more spread out and less tightly bound to the nucleus compared to sp^2^-hybridized carbons. Consequently, sp^3^-hybridized carbons have a higher polarizability and ϵ value. In the case of furan, the oxygen atom withdraws electrons from the ring, causing the π-electrons in the carbon atoms of furan to be more loosely held and more easily polarizable than those in benzene. As a result, the carbon atoms in furan exhibit a higher ϵ value.

For oxygen atoms, those connected to hard Lewis-acid metals exhibit lower polarizability and, therefore, a lower ϵ compared to oxygen in the original UFF. Since metals in group IIIA have higher charges than those in group IIA, the oxygen atoms bonded to group IIIA metals display a lower ϵ value. In this work, we provide a workflow to refit parameters when a significant change in the local chemical environment with different polarizability occurs. The refined FF may still work even if only a few atoms are in a different environment.

A summary of all the refined force field parameters we derived in this work is shown in Table 1. We would like to point the reader to Section 4 of the SI for a critical discussion of the physical relevance of these parameters.

**Table 1:** Refitted ϵ for Two Kinds of Oxygen and One Kind of Carbon^a^

atomic type	ϵ/*k*_B_ (K)	σ (Å)
O within M(IIIA)-MOFs	4.1	3.1181
O within M(IIA)-MOFs	11.9	3.1181
C within carboxyl ligands	34.7	3.4309

a The others are inherited from UFF.^5^

## 4 Concluding Remarks

A characteristic of the generic force fields (e.g., UFF and Dreiding) is that they use one set of Lennard-Jones parameters for each element. For many systems, this assumption works well. However, as shown in this work, MOFs have much richer local chemical environments than envisioned in developing the UFF force field. The consequence is that UFF shows systematic errors for some MOFs in predicting CO_2_ adsorption isotherms due to their specific chemical environments. To address this issue, we have introduced additional sets of Lennard-Jones parameters for these specific chemical environments, MOFs containing metals in group IIA and group IIIA, and carbons that are sp^3^ and sp^2^-hybridized. These parameters have been derived from fitting to experimental isotherms. We further validated these results by comparing them with binding energies computed from DFT.

We mainly develop force fields for the inaccuracy of UFF caused by different polarizability within different chemical environments, which was ignored in previous works when developing force fields for MOFs. The workflow described in the paper is a simple tool that can be used for systems in which the UFF needs to be tuned.

We use binding energies from DFT calculation to validate our refined force field for systems where we could not find experimental data. However, it is important to realize that even if the refined FF can reproduce experimental isotherms accurately, the binding energies from the refined FF do not agree perfectly with the DFT calculations. We typically observe differences up to 3.5 kJ mol^–1^. However, if these differences are even larger, it is unlikely that the FF can give an accurate description of the adsorption isotherm. This suggests we can use the difference between the FF and DFT binding energies as a diagnostic tool. If these differences are larger than 3.5 kJ mol^–1^, one needs to refine the force field to obtain accurate predictions of the CO_2_ isotherms.

## Supporting Information Available

List of modified structures; comparison between experimental isotherms and the simulated ones from the Dreiding force field; refined force field parameters obtained by tuning only Al or both Al and O; refined parameters derived from MIL-68(In); application of refined parameters to CAU-10-OCH_3_; and discussion on the uniqueness and physical relevance of the refined parameters (PDF)

## Notes

All structure files, simulation codes, and force field optimization scripts are available on Zenodo 10.5281/zenodo.13355868). The latest version of the workflow can be found on GitHub at https://github.com/legend-L24/ff_optimizer.
