# Supplementary material for: A Refined Set of Universal Force Field Parameters for Some Metal Nodes in Metal–Organic Frameworks
Source: J Chem Theory Comput. 2024 Nov 27;20(23):10540–52. doi: 10.1021/acs.jctc.4c01113 (PMC11635978; doi:10.1021/acs.jctc.4c01113)
Supplement: Supplementary file 1 — ct4c01113_si_001.pdf [file ct4c01113_si_001.pdf]

Supporting Information: A Refined Set of Universal Force Field Parameters for Some Metal Nodes in Metal-Organic Frameworks

Yutao Li, Xin Jin, Elias Moubarak, and Berend Smit*

Laboratory of molecular simulation (LSMO), Institut des Sciences et Ingénierie Chimiques, École Polytechnique Fédérale de Lausanne (EPFL), Rue de l'Industrie 17, CH-1951 Sion, Switzerland

E-mail: berend.smit@epfl.ch

Contents

1 Additional Force Fields **S-3**

1.1 Dreiding Force Field S-3

1.2 Tuning the Lennard-Jones parameters S-3

1.3 Uniqueness of force field parameters S-4

2 The List of Modified Structures **S-7**

3 Details of the fitting **S-8**

3.1 CAU-10-OCH3 . . . . . S-8

3.2 CAU-10(Al) and MIL-68(In) S-8

4 Comments of the reviewer **S-10**

References **S-12**

1 Additional Force Fields

1.1 Dreiding Force Field

Adsorption isotherms in MOFs have been predicted with both the UFF[S1] and the Dreiding[S2] force fields. In this work, we have focussed on the UFF force field. Figure S1 illustrates that the Dreiding force field, like UFF, systematically overestimates CO_2 uptake in MOFs containing hard Lewis acid metals.

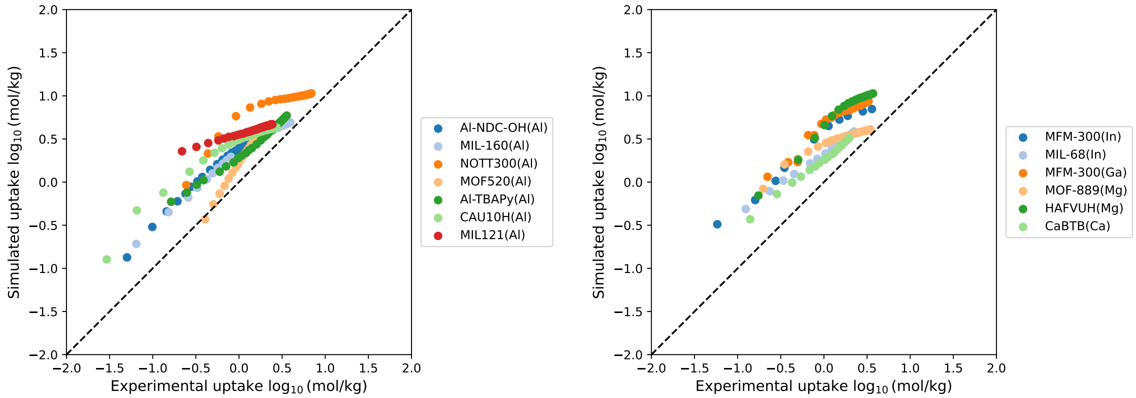

(a) (b)

Figure S1: (a) Comparison of isotherms from the Dreiding force field and experimental measurements for Al-MOFs.[S3–S9] (b) Comparison of isotherms from the Dreiding force field and experimental measurements for M-MOFs(M=Ga, In, Mg, and Ca).[S10–S15]

1.2 Tuning the Lennard-Jones parameters

As the UFF force field overestimates the adsorption for Al-MOFs, a logical first step is to optimize the Lennard-Jones interactions of CO_2 with the metal in the MOF. However, at the binding site, the interactions of CO_2 with the metal atoms are relatively small, and tuning the Al parameter did not give the desired results. Figure S2a compares the experimental CO_2 adsorption isotherms of CAU-10(Al) with the ones from the UFF and the tuned UFF in which we have set the interactions between CO_2 and Al to zero. Figure S2b compares the experimental adsorption isotherms of the other five Al-MOFs with the ones from the tuned

UFF.

If we tune both ϵ_O and ϵ_{Al} within metal clusters to correct the interaction between CO_2 and Al, we obtained $\epsilon_O/k_B = 1.9\,K$ and $\epsilon_{Al}/k_B = 5.1\,K$.

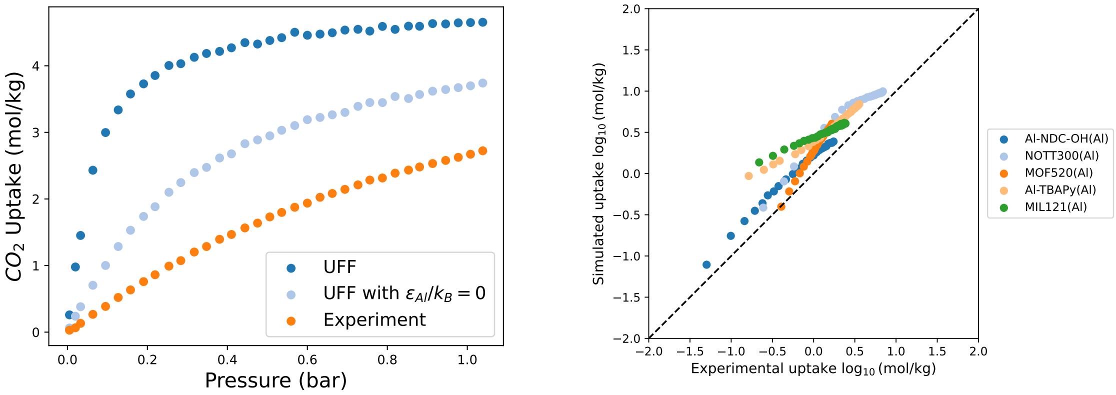

(a) (b)

Figure S2: (a) Comparison of the CO_2 isotherms at 296 K for CAU-10 from UFF, UFF with $\epsilon_{Al}/k_B = 0$ and experimental measurement. (b) Comparison of isotherms for the other five Al-MOFs from UFF with $\epsilon_{Al}/k_B = 0$, experimental measurement

1.3 Uniqueness of force field parameters

One can expect many different sets of parameters that give us a reasonable description of the isotherms. In the main text, we have already used CAU-10 as a starting point to obtain a refined set of UFF. In addition, we used CAU-10 to obtain the other 7 different sets of parameters in the range $\epsilon_C/k_B(K) \in [15.6, 39.5]$ and $\epsilon_O/k_B(K) \in [0.0, 44.41]$ shown in Figure S3a.

Firstly, we refined ϵ_C and ϵ_O at the same time and obtained three different FFs, including the one in the main text. The three sets are in the range $\epsilon_C/k_B(K) \in [27.7, 34.7]$ and $\epsilon_O/k_B(K) \in [4.1, 9.2]$, which are blue dots in Figure S3a. Furthermore, we can make a simple extrapolation of ϵ_C outside the range of the blue dots. We selected a few additional values of ϵ_C and determined the optimal value of ϵ_O (orange dots in Figure S3a). Together with the blue dots, this gives us eight different FFs.

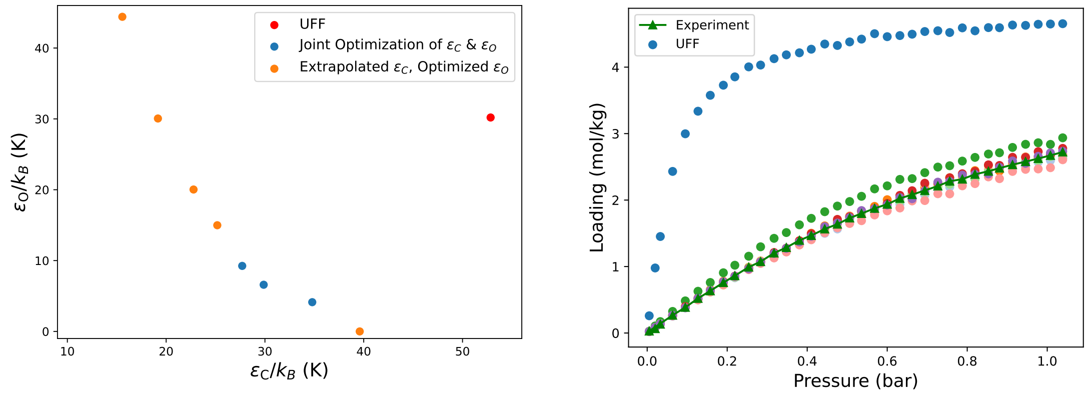

(a) (b)

Figure S3: (a) The plot of ϵ_C/k_B and ϵ_O/k_B values for UFF and eight refined force fields, refitted using the experimental isotherms of CAU-10. (b) A comparison between the experimental isotherm of CAU-10 and the simulated isotherms generated by UFF and the eight refined force fields.

All eight FFs can reproduce the whole experimental isotherm of CAU-10 shown in Figure S3b. To evaluate the transferability of these FFs, we applied them to five other Al-based MOFs: NOTT-300,[S3] MOF-520,[S4] Al-TBAPy,[S6] Al(NDC)(OH),[S7] and MIL-121.[S8] The average relative error of each point between the experimental and simulated isotherms was calculated using Equation (1). As shown in Figure S4a, FFs obtained through joint optimization (blue dots) demonstrated stronger transferability compared to those from extrapolation (orange dots). By simultaneously optimizing both ϵ_C and ϵ_O, we obtained a refined FF set that closely matches the experimental isotherms while maintaining minimal deviation from the UFF. This approach, illustrated in Figure S4a, yields FFs with superior transferability across different systems.

$$\text{Relative Error} = \frac{1}{M}\sum_{j=1}^{M}\left(\frac{1}{N_j}\sum_{i=1}^{N_j}\frac{|\mathrm{n}_{i,\mathrm{exp}}^{(j)} - \mathrm{n}_{i,\mathrm{sim}}^{(j)}|}{\mathrm{n}_{i,\mathrm{exp}}^{(j)}}\right), \quad (1)$$

where M is the total number of isotherms, N_j is the number of data points in the j-th isotherm, and $\mathrm{n}_{i,\mathrm{exp}}^{(j)}$ and $\mathrm{n}_{i,\mathrm{sim}}^{(j)}$ are the experimental and simulated values at the i-th data point of the j-th isotherm, respectively.

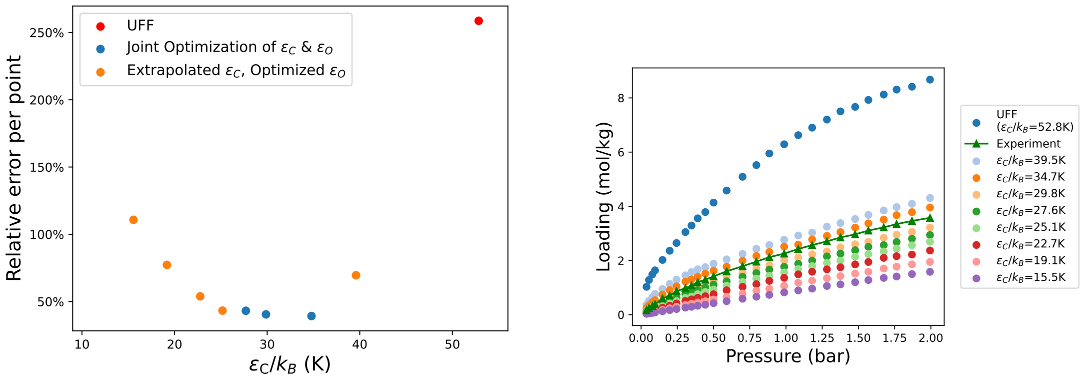

(a) (b)

Figure S4: (a) The plots of ϵ_C/k_B in UFF and 8 different FFs with their relative errors on five Al-MOFs. (b)A comparison between the experimental isotherm of Al-TBAPy and the simulated isotherms generated by UFF and the eight refined force fields.

To explain the different transferabilities of these FFs, we choose Al-TBAPy as an example because CO_2 is mainly attracted by ligands within it. We analysed the CO_2 binding sites of CAU-10 and Al-TBAPy from DFT optimization. In Table S1, we calculated the minimum distance between oxygen atoms in CO_2 and the framework atoms forming the binding sites. The adsorption site of Al-TBAPy is distant from the AlO_6 metal cluster, while the one of CAU-10 is close to both the metal cluster and ligand. We compared the experimental isotherm of Al-TBAPy with predictions from different FFs in Figure S4b. In Figure S4b and S3b, the refined set with $\epsilon_C/k_B = 34.7\,\mathrm{K}$ can produce experimental isotherms of both CAU-10 and Al-TBAPy. It means that the force field accurately describes the interactions of CO_2 with both the ligands and metal clusters separately.

Table S1: The minimum distances between oxygen atoms of CO_2 and different elements in MOFs.[S1]

Minimum distances between O(CO_2) and MOFs(Å)		
**element type**	**Al-TBAPy**	**CAU-10**
C	3.35	3.32
O	7.58	3.04
Al	9.26	4.12

2 The List of Modified Structures

Most of the structures we studied were taken from the PrISMa structures database.[S16] We modified the following two structures: MOF-520 (RSM4131.cif) and MIL-121 (RSM0112.cif). These structures were missing hydrogen atoms. We manually added the missing hydrogen atoms, and the *Cp2kMultistageDdec* work chain in the *aiida-lsmo* plugin was used to optimize the modified structures.

All structures mentioned in the article can be found on GitHub (`https://github.com/legend-L24/ff_optimizer`) or Zenodo (doi:10.5281/zenodo.13355868).

3 Details of the fitting

3.1 CAU-10-OCH3

We refitted the Lennard-Jones parameter ϵ_O using the experimental CO_2 adsorption isotherm data for CAU-10. We want to transfer this force field to CAU-10-OCH_3, where a single hydrogen atom in the benzene rings of CAU-10 is replaced by a OCH_3 functional group. Since oxygen atoms in the OCH_3 groups are not coordinated to Al, the question arises whether to use the newly refitted ϵ_O or the original UFF value for these oxygen atoms. As shown in Figure S5, both choices yield similar results, as most oxygen atoms still belong to carboxyl groups, coordinated to Al.

In Figure S5, the refined force field accurately reproduces the low-pressure region of the experimental isotherm for CAU-10-OCH_3, but it underestimates the loading at high pressures. In contrast, the refined force fields can reproduce the entire isotherm of CAU-10, as shown in the main text. For both CAU-10 and CAU-10-OCH_3, the MOF channels are relatively narrow, and the rotation of the OCH_3 groups in CAU-10-OCH_3 could increase the accessible volume for CO_2 adsorption. In our GCMC simulations, however, we assumed rigid frameworks, which may cause the observed underestimation of the CO_2 saturation loading in CAU-10-OCH_3.

3.2 CAU-10(Al) and MIL-68(In)

In our work, we systematically fitted the parameters to experimental data for one MOF and applied it to others with the same metal. We have selected CAU-10(Al) as our reference material. However, we could also select MIL-68(In). The difference between the force fields derived from CAU-10(Al) and MIL-68(In) is the ϵ_O/k_B, i.e., 4.1 K and 5.1 K, respectively.

Figure S6 shows that the two FFs generate almost the same isotherms for MIL-68(In). This suggests that we can transfer the FF for Al-MOFs to In-MOFs. Other examples in the main text also support this hypothesis.

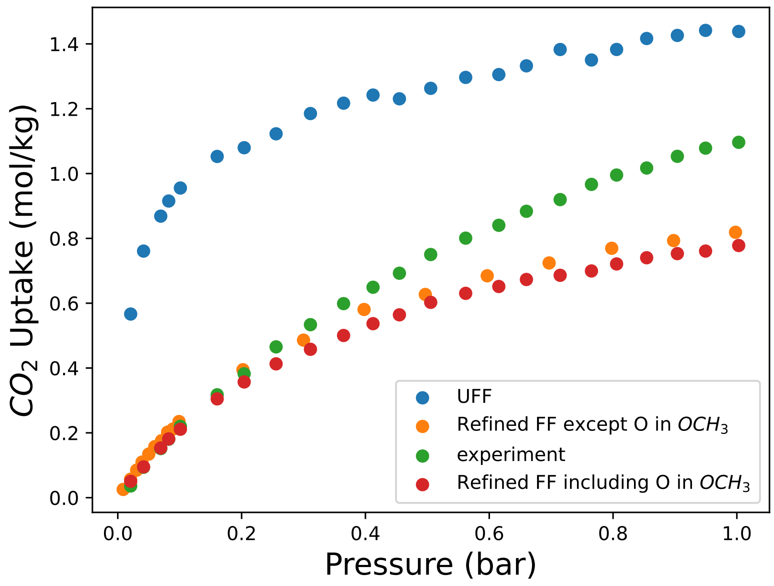

Figure S5: Comparison of isotherms for CAU-10-OCH_3 from UFF, experiments, and the refined FF with or without the specific parameter for oxygen of OCH_3 [S5]

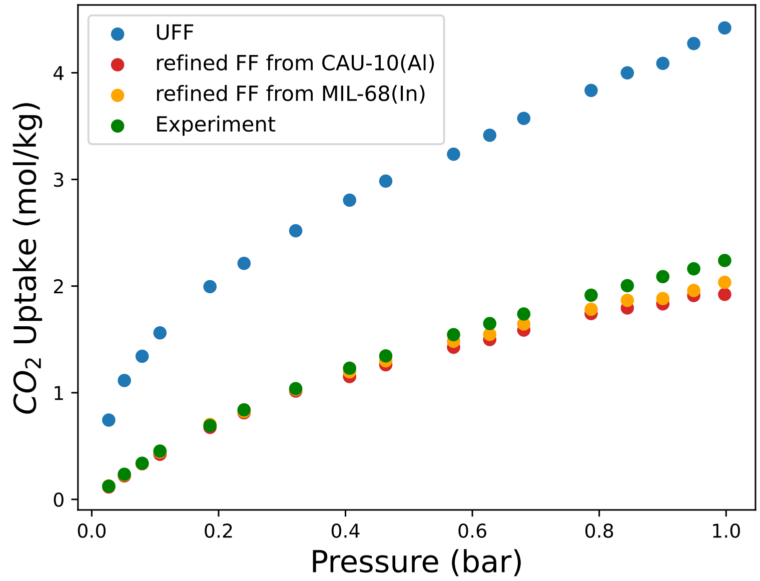

Figure S6: Comparison of isotherms for MIL-68(In)[S14] generated with the two force fields with experimental data. One FF was derived from experimental data for CAU-10(Al) (ϵ_O/k_B=4.1 K) and the other from data for MIL-68(In) (ϵ_O/k_B=5.1 K)

4 Comments of the reviewer

In our work, we have argued that our force field is a reasonable approach that addresses an important practical failure of the UFF force field to describe the interactions for some metals in MOFs correctly.

However, one of the reviewers of our work had fundamental criticism of our approach and work. We felt it to be important that this criticism is shared with the readers.

From the report of the reviewer (we corrected some typos)

> ... The authors present experimental data showing that the united force field (UFF) Lennard-Jones parameters do not have good accuracy for reproducing experimentally-measured gas adsorption isotherms in some metal-organic frameworks containing highly charged metal cations (e.g., Al, In, Ga, Mg, Ca). The authors propose a revised set of Lennard-Jones parameters for oxygen and carbon atoms in these MOFs to address this problem so that the computed adsorption isotherms using the revised parameters give good agreement with the experimentally-measured isotherms. However, close examination reveals a fundamental problem with their revised parameter values.
>
> Even though their revised Lennard-Jones parameters give computed adsorption isotherms in good agreement with the experimentally-measured gas adsorption isotherms in these MOFs, their revised Lennard-Jones parameters are nonetheless physically implausible for the following two reasons: (1) Their revised Lennard-Jones parameters for "O within M(IIIA)-MOFs" epsilon LJ = 4.1 K is much too small, and (2) They retained the original UFF epsilon LJ = 0.505 kcal/mol = 254 K for the Al(III) atom, which is much too large. How can this picture be reconciled in which the revised LJ parameter values were assigned physically implausible values yet gave computed gas adsorption isotherms in good agreement with experimental values?

This can be interpreted as the effects of multicollinearity in the parameter optimization landscape. For optimization problems experiencing multicollinearity, more than one set of distinct parameter values can yield small loss function values. In these cases, simply achieving a small value of the loss function does not necessarily yield physically-realistic parameter values. In other words, it may be possible to reproduce the experimentally-measured gas adsorption isotherms using different sets of LJ parameter values. (Artificially reducing the number of regression parameters, such as choosing only to re-optimize LJ parameters on oxygen and carbon atoms instead of all atoms in the MOF is *not* a good approach to this multicollinearity problem!

Consequently, simply optimizing the LJ parameter values to reproduce the experimentally measured gas adsorption isotherms does not necessarily yield physically reasonable LJ parameter values. How to resolve this multicollinearity problem? The multicollinearity problem could potentially be treated using an optimization strategy that incorporates some physical information about each atom in the material. For example, the loss function could be recast so that the LJ parameters are optimized to reproduce the experimentally-measured adsorption isotherms subject to a regularization penalty term (r.p.t.) that constrains the optimized parameter values to some physically realistic subdomain of the parameter space. Various strategies for constructing the r.p.t. could be contemplated. This would require some careful thought and would be an extensive endeavor that would take substantial time to complete. . . .

References

(S1) Rappé, A. K.; Casewit, C. J.; Colwell, K.; Goddard III, W. A.; Skiff, W. M. UFF, a full periodic table force field for molecular mechanics and molecular dynamics simulations. *Journal of the American chemical society* **1992**, *114*, 10024–10035.

(S2) Mayo, S. L.; Olafson, B. D.; Goddard, W. A. Dreiding - a Generic Force-Field for Molecular Simulations. *J Phys Chem-Us* **1990**, *94*, 8897–8909.

(S3) Yang, S.; Sun, J.; Ramirez-Cuesta, A. J.; Callear, S. K.; David, W. I.; Anderson, D. P.; Newby, R.; Blake, A. J.; Parker, J. E.; Tang, C. C.; others Selectivity and direct visualization of carbon dioxide and sulfur dioxide in a decorated porous host. *Nature chemistry* **2012**, *4*, 887–894.

(S4) Wan, Y.; Miao, Y.; Zhong, R.; Zou, R. High-selective CO_2 capture in amine-decorated Al-MOFs. *Nanomaterials* **2022**, *12*, 4056.

(S5) Reinsch, H.; van der Veen, M. A.; Gil, B.; Marszalek, B.; Verbiest, T.; De Vos, D.; Stock, N. Structures, sorption characteristics, and nonlinear optical properties of a new series of highly stable aluminum MOFs. *Chemistry of Materials* **2013**, *25*, 17–26.

(S6) Boyd, P. G.; Chidambaram, A.; García-Díez, E.; Ireland, C. P.; Daff, T. D.; Bounds, R.; Gładysiak, A.; Schouwink, P.; Moosavi, S. M.; Maroto-Valer, M. M.; others Data-driven design of metal–organic frameworks for wet flue gas CO2 capture. *Nature* **2019**, *576*, 253–256.

(S7) Zhang, J.; Sun, L.; Xu, F.; Li, F.; Zhou, H.-Y.; Liu, Y.-L.; Gabelica, Z.; Schick, C. H_2 storage and CO_2 capture on a nanoscale metal–organic framework with high thermal stability. *Chemical Communications* **2012**, *48*, 759–761.

(S8) Volkringer, C.; Loiseau, T.; Guillou, N.; Férey, G.; Haouas, M.; Taulelle, F.; Elkaim, E.; Stock, N. High-throughput aided synthesis of the porous metal–organic

framework-type aluminum pyromellitic, MIL-121, with extra carboxylic acid functionalization. *Inorganic chemistry* **2010**, *49*, 9852–9862.

(S9) Pei, J.; Wen, H.-M.; Gu, X.-W.; Qian, Q.-L.; Yang, Y.; Cui, Y.; Li, B.; Chen, B.; Qian, G. Dense Packing of Acetylene in a Stable and Low-Cost Metal–Organic Framework for Efficient C_2H_2/CO_2 Separation. *Angewandte Chemie International Edition* **2021**, *60*, 25068–25074.

(S10) Nguyen, P. T.; Nguyen, H. T.; Pham, H. Q.; Kim, J.; Cordova, K. E.; Furukawa, H. Synthesis and selective CO_2 capture properties of a series of hexatopic linker-based metal–organic frameworks. *Inorganic Chemistry* **2015**, *54*, 10065–10072.

(S11) Noh, K.; Ko, N.; Park, H. J.; Park, S.; Kim, J. Two porous metal–organic frameworks containing zinc–calcium clusters and calcium cluster chains. *CrystEngComm* **2014**, *16*, 8664–8668.

(S12) Lin, Q.; Wu, T.; Zheng, S.-T.; Bu, X.; Feng, P. A chiral tetragonal magnesium-carboxylate framework with nanotubular channels. *Chemical Communications* **2011**, *47*, 11852–11854.

(S13) Savage, M.; Cheng, Y.; Easun, T. L.; Eyley, J. E.; Argent, S. P.; Warren, M. R.; Lewis, W.; Murray, C.; Tang, C. C.; Frogley, M. D.; others Selective adsorption of sulfur dioxide in a robust metal–organic framework material. *Advanced materials* **2016**, *28*, 8705–8711.

(S14) Wu, L.; Wang, W.; Liu, R.; Wu, G.; Chen, H. Impact of the functionalization onto structure transformation and gas adsorption of MIL-68 (In). *Royal Society Open Science* **2018**, *5*, 181378.

(S15) Krap, C. P.; Newby, R.; Dhakshinamoorthy, A.; García, H.; Cebula, I.; Easun, T. L.; Savage, M.; Eyley, J. E.; Gao, S.; Blake, A. J.; others Enhancement of CO_2 adsorption

and catalytic properties by Fe-doping of $Ga_2(OH)_2(L)(H_4L$ = biphenyl-3, 3′, 5, 5′-tetracarboxylic acid), MFM-300 (Ga_2). *Inorganic chemistry* **2016**, *55*, 1076–1088.

(S16) Charalambous, C.; Moubarak, E.; Schilling, J.; Sanchez Fernandez, E.; Wang, J.-Y.; Herraiz, L.; Mcilwaine, F.; Peh, S. B.; Garvin, M.; Jablonka, K. M.; others A holistic platform for accelerating sorbent-based carbon capture. *Nature* **2024**, 1–6.
